# Capicua regulates the survival of Cajal-Retzius cells in the postnatal hippocampus

**DOI:** 10.1038/s41419-025-08206-7

**Published:** 2025-12-22

**Authors:** Zain H. Patel, Rebekah van Bruggen, Mi Wang, Qiumin Tan

**Affiliations:** Department of Cell Biology, University of Alberta, Edmonton, AB Canada

**Keywords:** Cell death in the nervous system, Neuronal development

## Abstract

Programmed cell death is crucial for organ morphogenesis and tissue homeostasis. Understanding programmed cell death in the developing brain is essential for comprehending both normal brain development and neurological disorders. In this study, we utilize Cajal-Retzius (CR) cells, transient neurons that populate the embryonic cortex and are predominantly eliminated in early postnatal stages, as a model to investigate the regulation of programmed cell death. While many CR cells typically undergo postnatal cell death, some persist into adulthood in the hippocampus, influencing local circuits and behaviors. Here, we show that the loss of capicua (CIC), a transcriptional repressor implicated in a rare neurodevelopmental syndrome and multiple cancers, results in aberrant survival of CR cells in the adult hippocampus. Altered cell survival is mediated by the cell-autonomous function of CIC in hippocampal CR cells. Surprisingly, the atypical persistence of CR cells following CIC loss does not impact hippocampal-dependent behaviors or susceptibility to kainic acid-induced seizures. Single-cell transcriptomic analysis unveils previously unrecognized heterogeneity among hippocampal CR cells and suggests a role of CIC in repressing *Fgf1* expression. Additionally, we reveal that FGF1 and BCL2 serve as pivotal regulators enhancing CR cell survival in the postnatal hippocampus. Our findings shed light on a previously unacknowledged role of CIC upstream of FGF signaling and elucidate the apoptosis mechanism governing developmental programmed CR cell death.

## Introduction

In the developing cerebral cortex, a significant portion of newborn neurons undergo cell death [1, 2]. Programmed cell death in the developing nervous system serves to promote morphogenesis, shape signaling centers, and prune neurons that are targeting inappropriate regions [1, 3]. The regulation of programmed cell death and its influence on the cellular architecture of the cerebral cortex carry significant implications for normal brain development as well as neurological disorders, and yet, our understanding of this process is incomplete. Cajal-Retzius (CR) cells—pioneer neurons that populate the outer layer of the embryonic cortex but then disappear during early postnatal life—present a unique system to address this question. CR cells are a class of early-born, transient neurons that are abundant in the marginal zone of the cortex and the hippocampal primordium in the embryonic brain. CR cells are organizers of early cortical development as they regulate radial migration and laminar positioning of excitatory neurons [4–6]. Hippocampal CR cells arise from the cortical hem [6–11]. In contrast to their neocortical counterparts, which undergo rapid cell death in the first two postnatal weeks in rodents, a substantial proportion of hippocampal CR cells remain throughout adulthood [12–14]. These persistent hippocampal CR cells regulate the local circuit and related behaviors [12, 15–18]. As such, CR cell clearance and survival are tightly controlled. Indeed, abnormal CR cell persistence due to *Bax* deletion or PI3K/AKT/mTOR pathway activation leads to impaired learning and memory and/or heightened seizure susceptibility in mouse models [19, 20]. Likewise, previous studies have suggested a link between abnormal CR cell persistence in the hippocampus and epilepsy pathology in humans [21, 22]. Despite the need to tightly regulate CR cell death during development, the underlying mechanisms and signaling pathways remain elusive.

Identifying genes associated with neurodevelopmental disorders often provides unique insights into previously unrecognized mechanisms that regulate brain development. One such gene is capicua (*CIC*), a transcriptional repressor widely studied as a downstream effector of the RAS/MAPK signaling pathway in the context of *Drosophila* development and human cancers. Activation of growth factor or the RAS/MAPK signaling leads to CIC degradation or translocation from the nucleus to the cytoplasm, resulting in de-repression of CIC target genes such as the PEA3 family transcription factors *ETV4* and *ETV5* [23–27], which play critical roles in cell proliferation and survival. It is therefore not surprising that somatic *CIC* loss-of-function variants have been identified in many cancers [24, 26]. On the other hand, germline heterozygous loss-of-function *CIC* variants are associated with a rare neurodevelopmental disorder characterized by developmental delay, intellectual disability, hyperactivity, autism, and epilepsy [28–33]. While we have previously shown that CIC is broadly expressed throughout the brain, including the hippocampus [28, 34], the functional role of CIC in specific neuronal subtypes remains elusive. Here, we investigate the consequences of selective deletion of CIC from hippocampal CR cells and the signaling pathways that regulate CR cell death in the postnatal hippocampus.

## Results

### Capicua is required in Cajal-Retzius neurons to promote their developmental cell death

In mice, CR cell neurogenesis peaks between embryonic day (E)10.5 and E13.5 [35, 36]. By E12.5, CR cells are already present at the cortical hem—the major source of hippocampal CR cells—and begin migrating tangentially along the marginal zone (layer I in neocortex, molecular layer in hippocampus). CR cells become post-mitotic almost immediately after leaving the ventricular zone of the hem at E13.5 [37]. Thus, they constitute a post-mitotic population from early embryogenesis onward, with no appreciable neurogenesis occurring after birth. Instead, CR cells undergo massive programmed cell death in the postnatal brain: between postnatal day (P)8 and P20, approximately 70% of hippocampal CR cells are eliminated [13, 14].

**Fig. 1:**
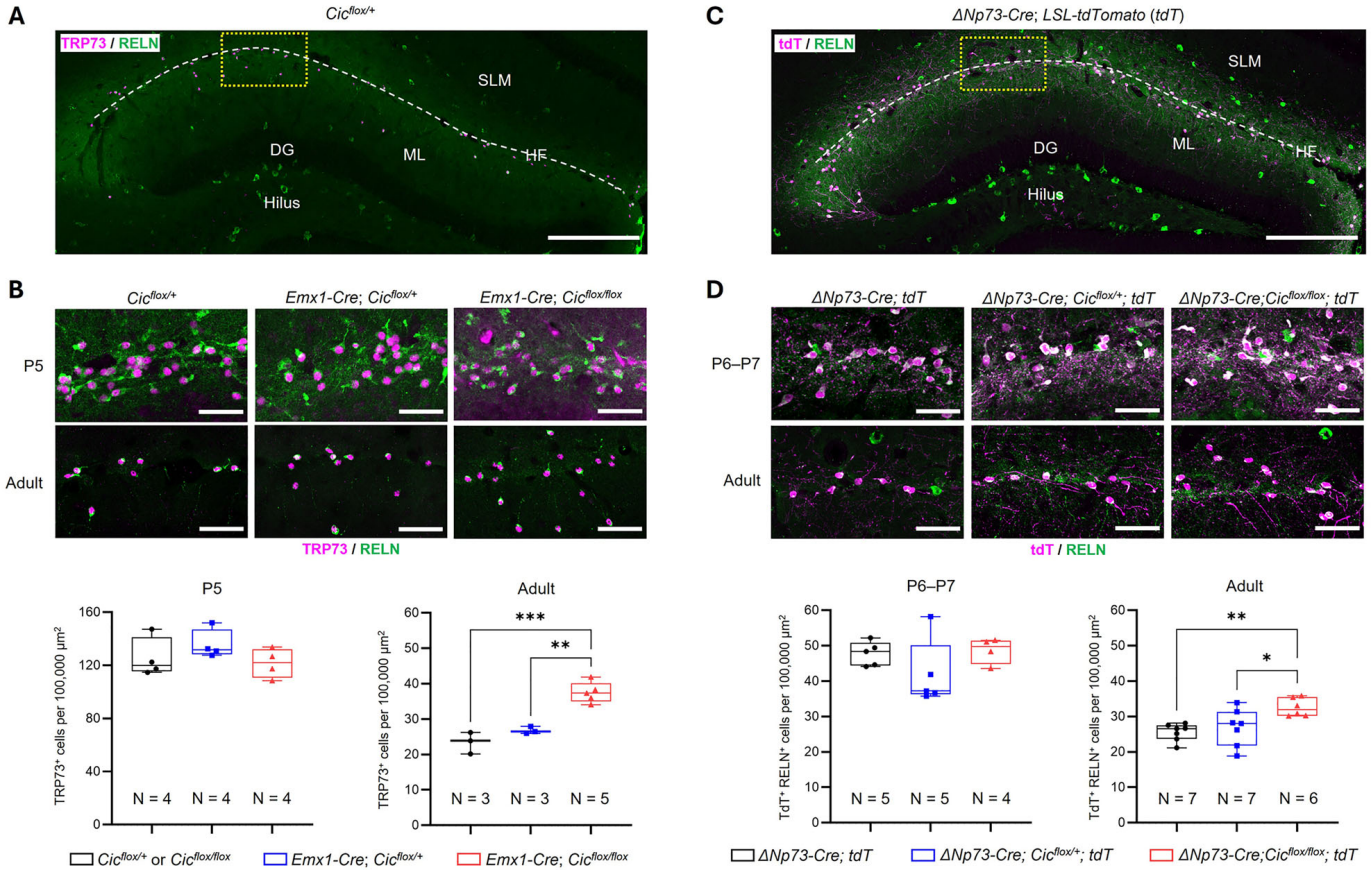
Loss of CIC leads to abnormal persistence of Cajal-Retzius cells in the adult hippocampus. **A** Representative overview of a coronal section of the dentate gyrus in the adult *Cic*^*flox/+*^ mouse hippocampus. Scale bar = 250 µm. **B** *Top:* Representative images of CR cells, identified by TRP73 and RELN immunostaining, along the hippocampal fissure from five-day old (P5) and adult aged (11-week-old) *Cic*^*flox/+*^, *Emx1-Cre; Cic*^*flox/+*^ and *Emx1-Cre; Cic*^*flox/flox*^ mice. Scale bars = 50 µm. *Bottom*: Quantification of CR cell density along the hippocampal fissure. **C** Representative overview of a coronal section of the dentate gyrus in the adult *ΔNp73-Cre; tdT* mouse. Scale bar = 250 µm. **D** *Top:* Representative images of CR cells identified by tdT and RELN immunostaining along the hippocampal fissure, from P6–P7 and adult (7-week-old) *ΔNp73-Cre; tdT*, *ΔNp73-Cre; Cic*^*flox/+*^*; tdT*, and *ΔNp73-Cre; Cic*^*flox/flox*^*; tdT* mice. Scale bars = 50 µm. *Bottom*: Quantification of CR cell density along the hippocampal fissure. SLM stratum lacunosum-molecular, HF hippocampal fissure, DG dentate gyrus, ML molecular layer. Data are presented as box and whiskers plots with all data points shown. Each data point represents a single animal, wherein three coronal sections of the hippocampus were quantified and averaged. Statistical analysis was performed using one-way ANOVA followed by Tukey’s *post hoc* test. *, *p* < 0.05; **, *p* < 0.01; ***, *p* < 0.001.

To address the role of CIC in hippocampal CR cells, we first utilized *Emx1-Cre; Cic*^*flox/flox*^ mice, in which Cre is activated in progenitor cells that give rise to forebrain excitatory neurons (including hem-derived CR cells) and glia beginning at E9.5 [38]. As a result, *Cic* is deleted from hippocampal CR cells (Fig. S1A). To determine if CIC deletion alters the developmental cell death of CR cells, we compared the number of hippocampal CR cells between the *Emx1-Cre; Cic*^*flox/flox*^ mice and the *Cic*^*flox*^ control and conditional heterozygous *Emx1-Cre; Cic*^*flox/+*^ mice. Using the established CR cell markers, TRP73 and RELN, we found that at P5 the density of CR cells was similar across all three groups (Fig. 1A B), indicating that early CR cell development from progenitors was not affected by CIC deletion. To determine whether apoptotic cell death was altered in the knockout, we performed TUNEL staining (Fig. S2A). At P14, during the peak period of CR cell elimination [13, 14], we did not detect any TUNEL^+^ signals in RELN^+^ CR cells in either control or knockout hippocampi (Fig. S2B), possibly because CR cells lose RELN immunoreactivity as they undergo apoptosis. Nonetheless, the overall number of TUNEL^+^ cells along the hippocampal fissure was significantly reduced in *Emx1-Cre; Cic*^*flox/flox*^ mice (Fig. S2C). Consistent with this, there was a greater number of CR cells in the adult *Emx1-Cre; Cic*^*flox/flox*^ mouse hippocampus (Fig. 1B). Together, these results suggest that CIC does not affect the early generation of CR cells but is required for their normal postnatal elimination.

Besides CR cells, the *Emx1-Cre* mouse line also drives Cre-mediated recombination in other forebrain excitatory neurons [38]. This brought into question whether abnormal persistence of hippocampal CR cells in the *Emx1-Cre; Cic*^*flox/flox*^ mice was due to loss of CIC from CR cells or other excitatory neurons in the hippocampus. To address this, we turned to the *ΔNp73-Cre* mouse line, which initiates Cre expression in CR cell progenitors at E11.5 and remains largely restricted to these cells during early postnatal stages [14, 39]. Additionally, we incorporated the Cre-dependent reporter LSL-tdTomato (*tdT*) to generate the *ΔNp73-Cre; Cic*^*flox/flox*^*; tdT* mice. We first characterized the knockout efficiency of the *ΔNp73-Cre; Cic*^*flox/flox*^*; tdT* mouse line and found that CIC was deleted from approximately 75% of CR cells in the hippocampus (Fig. S1B). At P5, short-term EdU (5-ethynyl-2´-deoxyuridine) labeling confirmed that all hippocampal CR cells were post-mitotic in both control and knockout mice (Fig. S2), and CR cell density at around P6 was similar between the genotypes (Figs. 1C, D and S3C). At P14, during the peak period of CR cell death, TUNEL staining revealed a significant reduction in apoptotic cells in *ΔNp73-Cre; Cic*^*flox/flox*^ mice (Fig. S2D). Consistent with this, CR cell density was significantly greater in the *ΔNp73-Cre; Cic*^*flox/flox*^*; tdT* adult hippocampus (Fig. 1D). Together, these findings indicate that CIC is not required for the generation or early maturation of CR cells but acts cell-autonomously within CR cells to ensure their normal developmental elimination in the postnatal hippocampus.

### Loss of CIC from CR cells does not affect cognitive behaviors and seizure susceptibility

**Fig. 2:**
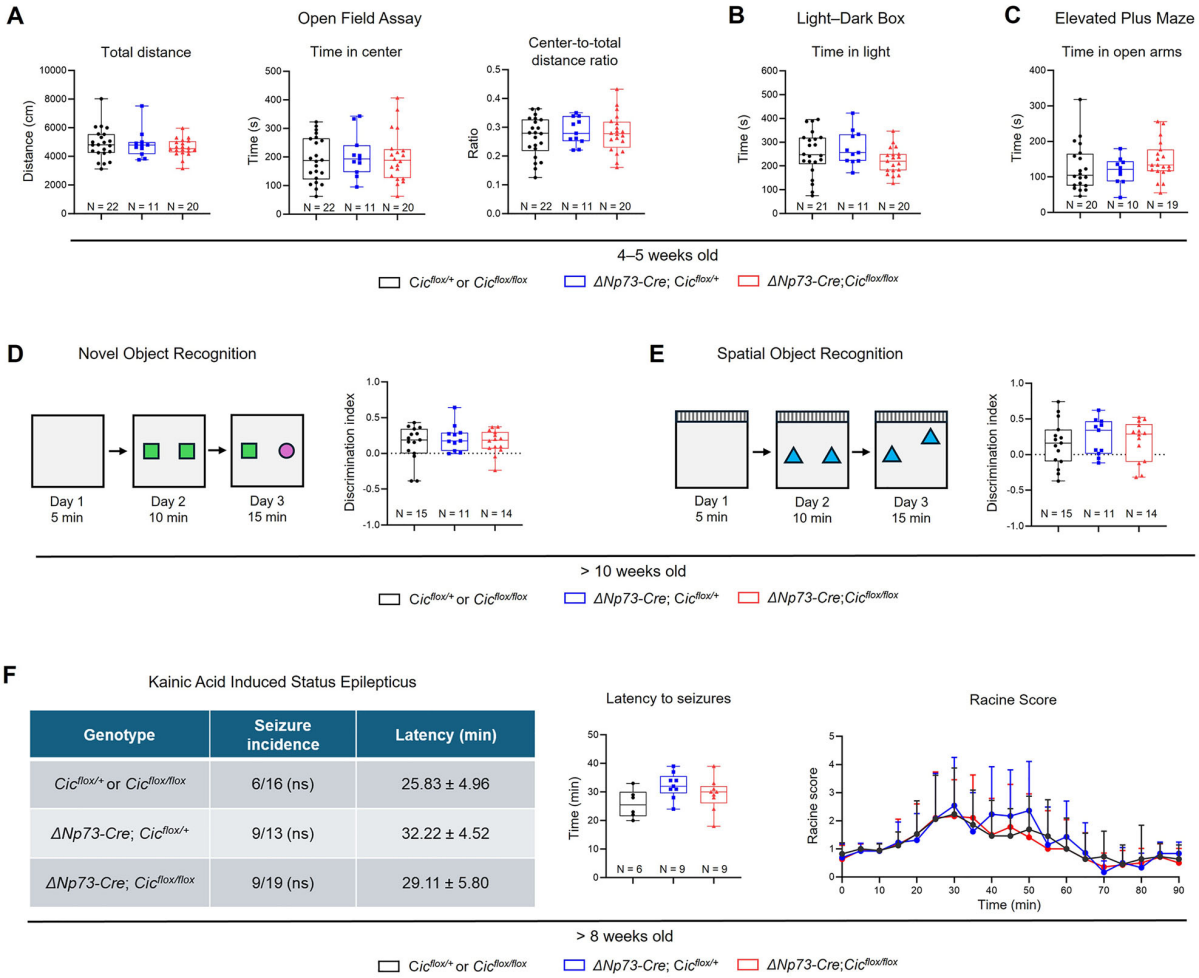
Selective loss of CIC from Cajal-Retzius cells does not affect cognitive function or kainic acid-induced seizure susceptibility. 4- to 5-week-old *Cic*^*flox*^, *ΔNp73-Cre; Cic*^*flox/+*^, and *ΔNp73-Cre; Cic*^*flox/flox*^ mice were subject to **A** the open field assay, **B** the light–dark box assay, and (**C**) the elevated plus maze test. **D** 10-week or older *Cic*^*flox*^, *ΔNp73-Cre; Cic*^*flox/+*^, and *ΔNp73-Cre; Cic*^*flox/flox*^ mice were subject to the novel object recognition assay. *Left:* Overview of the novel object recognition paradigm. *Right*: Quantification of the discrimination index. A positive value indicates the mouse spent more time with the novel object compared to the familiar object. **E** 10-week or older mice were subject to the spatial object recognition assay. *Left:* Overview of the experimental paradigm. *Right*: Quantification of the discrimination index. A positive value indicates the mouse spent more time with the moved object compared to the static object. **F** 8-week or older mice were intraperitoneally injected with 20 mg/kg of kainic acid. Mice were monitored for 90 minutes, and behavioral seizures were scored according to a modified Racine scale. *Left:* Summary of the number of mice that developed seizures in each group and the latency to seizure onset. *Middle*: Latency to seizure onset of the mice that developed a seizure. *Right*: Racine score over the 90-minute monitoring period. Data are presented as box and whisker plots with all data points shown, or as a line graph with error bars representing ± SD. Statistical analysis was performed using one-way ANOVA followed by Tukey’s *post hoc* test. Each data point represents a single animal.

CR cell-dependent glutamate transmission has recently been shown to be necessary for hippocampal-dependent behaviors, including anxiety and memory in young mice between 4–5 weeks of age [18]. We therefore tested if abnormal CR cell persistence due to the loss of CIC affects neurobehaviors in young mice. Four- to 5-week-old *ΔNp73-Cre; Cic*^*flox/flox*^ and control mice were subjected to behavioral analyses. In the open field assay, we did not observe any significant differences in distance traveled, time spent in the center of the arena, nor the center-to-total distance ratio (Fig. 2A), indicating normal locomotor activity in the mutant mice. In the light–dark box assay, the *ΔNp73-Cre; Cic*^*flox/flox*^ mice showed no differences in preference for the light region versus the dark region compared to the control mice (Fig. 2B). In the elevated plus maze test, the *ΔNp73-Cre; Cic*^*flox/flox*^ mice spent a similar amount of time in the open arms compared to the controls (Fig. 2C). These results suggest that anxiety-like behaviors were not changed in the knockout mice. The spontaneous Y maze test was further conducted to assess spatial working memory, and we found that the *ΔNp73-Cre; Cic*^*flox/flox*^ mice did not exhibit a significant difference in the alternation index (Fig. S4A).

Aberrant survival of CR cells due to *Bax* deletion impairs learning and memory in adult mice [20]. We therefore tested whether abnormal CR cell persistence due to the loss of CIC impairs cognitive functions in adult mice that are older than 8 weeks of age. We conducted novel object recognition and spatial object recognition assays to assess episodic memory and hippocampal-dependent spatial learning and memory, respectively. The discrimination index was calculated to determine the preference of a mouse for the novel object compared to the familiar object in the novel object recognition test, or the moved object compared to the static object in the spatial object recognition test. In both assays, there was no significant difference in the discrimination index between the *ΔNp73-Cre; Cic*^*flox/flox*^ mice and the controls (Fig. 2D, E). Additionally, we performed the open field assay and the elevated plus maze test and did not observe any differences in any of the parameters measured (Fig. S4B, C). We also found that mutant mice spent a similar amount of time freezing compared to the control groups in both the context- and cue-dependent fear conditioning assays (Fig. S4D).

As aberrant CR cell survival in the hippocampus is associated with increased seizure susceptibility [19, 20], we sought to determine whether abnormal CR cell persistence upon the loss of CIC affects kainic acid-induced seizures. To this end, adult control and *ΔNp73-Cre; Cic*^*flox/flox*^ mice received an intraperitoneal injection of kainic acid, and their behavior and seizure intensity were scored using the Racine scale over a 90-min period. There were no significant differences in the number of mice that developed seizures in each group, nor the latency to the first seizure (Fig. 2F). Additionally, the Racine scores in mice that developed seizures were similar across the three groups (Fig. 2F). These behavioral results demonstrate that ablation of CIC from CR cells does not negatively impact cognitive behaviors and kainic acid-induced seizures.

### BCL2 promotes hippocampal Cajal-Retzius cell survival

**Fig. 3:**
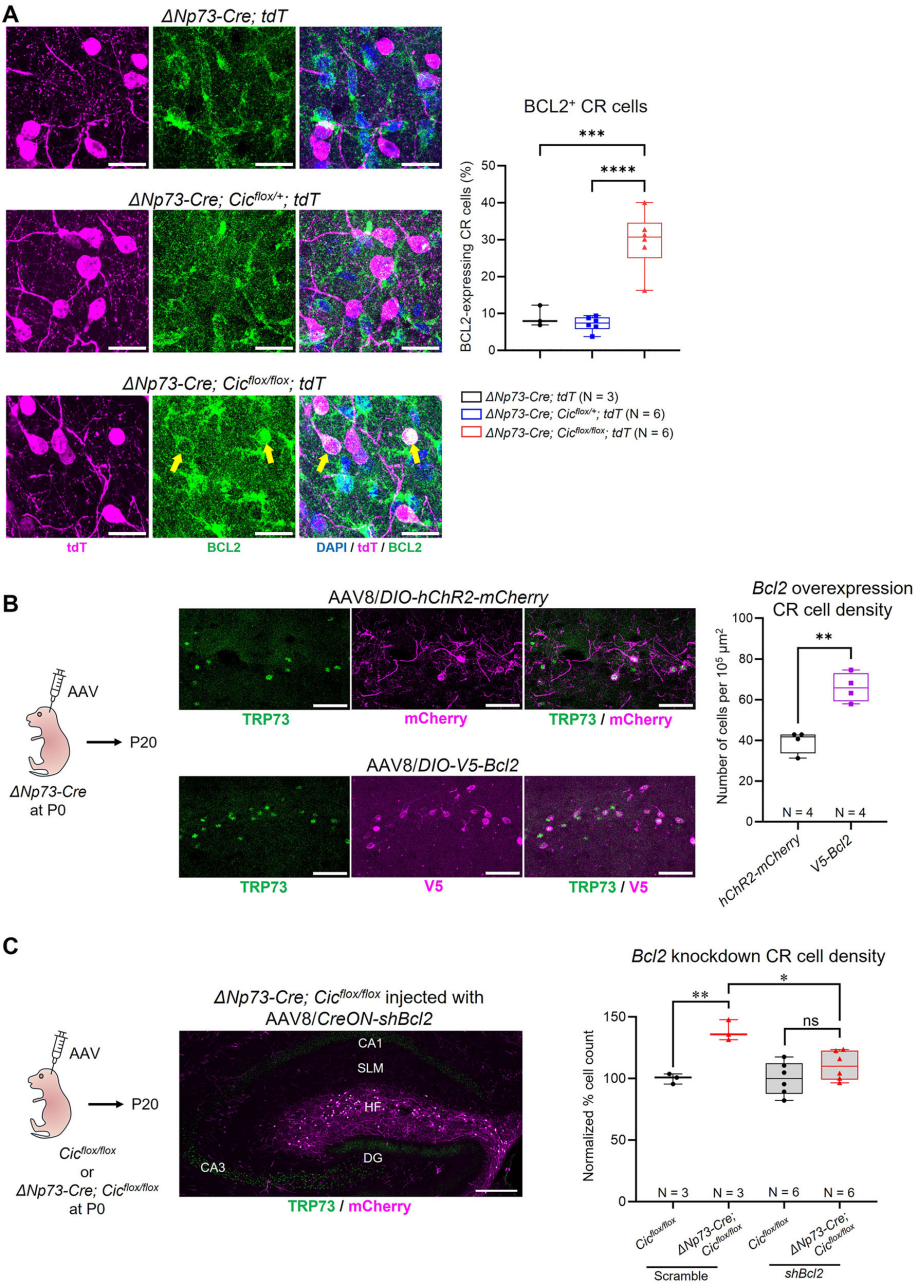
BCL2 promotes CR cell survival in the postnatal mouse hippocampus. **A** A greater proportion of CR cells express BCL2 in the *ΔNp73-Cre; Cic*^*flox/flox*^*; tdT* mouse hippocampus at P17. *Left*: Representative images of CR cells along the hippocampal fissure. CR cells were identified by their expression of tdTomato (tdT). Yellow arrows point to CR cells expressing BCL2 in the knockout mice. Quantification is shown on the right. Scale bars = 20 µm. Each data point represents a single animal wherein three coronal sections of the hippocampus were quantified and averaged. Statistical analysis was performed using one-way ANOVA followed by Tukey’s *post hoc* test. **B** *Left*: Schematic of the intracerebroventricular injection paradigm and experimental timeline. *Middle*: Representative images of CR cells along the hippocampal fissure in P20 *ΔNp73-Cre* mice injected with either AAV8/*DIO-hChR2-mCherry* or AAV8/*DIO-V5-Bcl2*. Scale bars = 50 µm. *Right*: Quantification of CR cell density along the hippocampal fissure. Each data point represents a single animal wherein three coronal sections of the hippocampus were quantified and averaged. Statistical analysis was performed using Welch’s *t*-test. **C** *Left*: Schematic of the intracerebroventricular injection paradigm and experimental timeline. *Middle*: Overview of the hippocampus from a P20 *ΔNp73-Cre; Cic*^*flox/flox*^ mouse injected with AAV8/*CreON-shBcl2*. SLM, stratum lacunosum-moleculare; HF, hippocampal fissure; DG, dentate gyrus. Scale bar = 200 µm. *Right*: Quantification of normalized CR cell density along the hippocampal fissure in P20 mice. Data from knockout mice were normalized to littermate controls injected with the same virus to minimize variation between litters and viral preparations. Statistical analysis was performed using nested one-way ANOVA followed by Sidak’s *post hoc* test, with three brain sections analyzed per animal and sections treated as nested observations. Each data point represents one animal. Data are presented as box and whisker plots with all data points shown. *, *p* < 0.05; **, *p* < 0.01; ***, *p* < 0.001; ****, *p* < 0.0001; ns, not significant.

Hippocampal CR cell death is partly dependent on the pro-apoptotic factor BAX [20], the function of which is sequestered by BCL2 [40–42]. Because the role of BCL2 has not been studied in the context of hippocampal CR cell death, we decided to further elucidate its role in this process. We first examined whether BCL2 levels were altered in the *ΔNp73-Cre; Cic*^*flox/flox*^*; tdT* knockout mice. We found that at P17, when hippocampal CR cells undergo rapid cell death [13, 14], a significantly larger proportion of CR cells in the knockout mice showed detectable levels of BCL2 compared to the controls (Fig. 3A). We next determined if overexpression of BCL2 was sufficient to drive abnormal CR cell persistence using adeno associated virus (AAV) carrying a Cre-dependent V5-tagged *Bcl2* construct (AAV8/*DIO-Bcl2-V5*) via intracerebroventricular injection into P0 *ΔNp73-Cre* pups (Figs. 3B and S5A). This approach efficiently transduces postnatal hippocampal CR cells without impacting other hippocampal neurons [14]. Control groups included wildtype pups injected with the same virus or *ΔNp73-Cre* pups injected with AAV8 carrying a Cre-dependent channelrhodopsin construct (AAV8/*DIO-hChR2-mCherry*). Quantification revealed significantly fewer TUNEL^+^ apoptotic cells at P14 and increased CR cell numbers at P20 in the hippocampal fissure of AAV8/*DIO-Bcl2-V5* injected mice compared to controls (Figs. 3B and S5B), demonstrating a pro-survival effect of BCL2 on hippocampal CR cells. To determine whether BCL2 mediates enhanced CR cell survival in the absence of CIC, we generated two Cre-dependent AAV/shRNA constructs targeting *Bcl2* (Fig. S6). Both constructs efficiently reduced BCL2 in vitro (Fig. S6C, E); however, only the U6 promoter-based AAV8/*CreON-shBcl2* significantly decreased CR cell density in *ΔNp73-Cre; Cic*^*flox/flox*^ mice, restoring it to control levels (Figs. 3C and S6F). Together, these findings demonstrate that BCL2 upregulation is both sufficient to promote CR cell survival and necessary for their abnormal persistence when CIC is deleted.

### Single-cell transcriptomics reveals distinct subtypes of *ΔNp73*-lineage Cajal-Retzius cells in the postnatal hippocampus

**Fig. 4:**
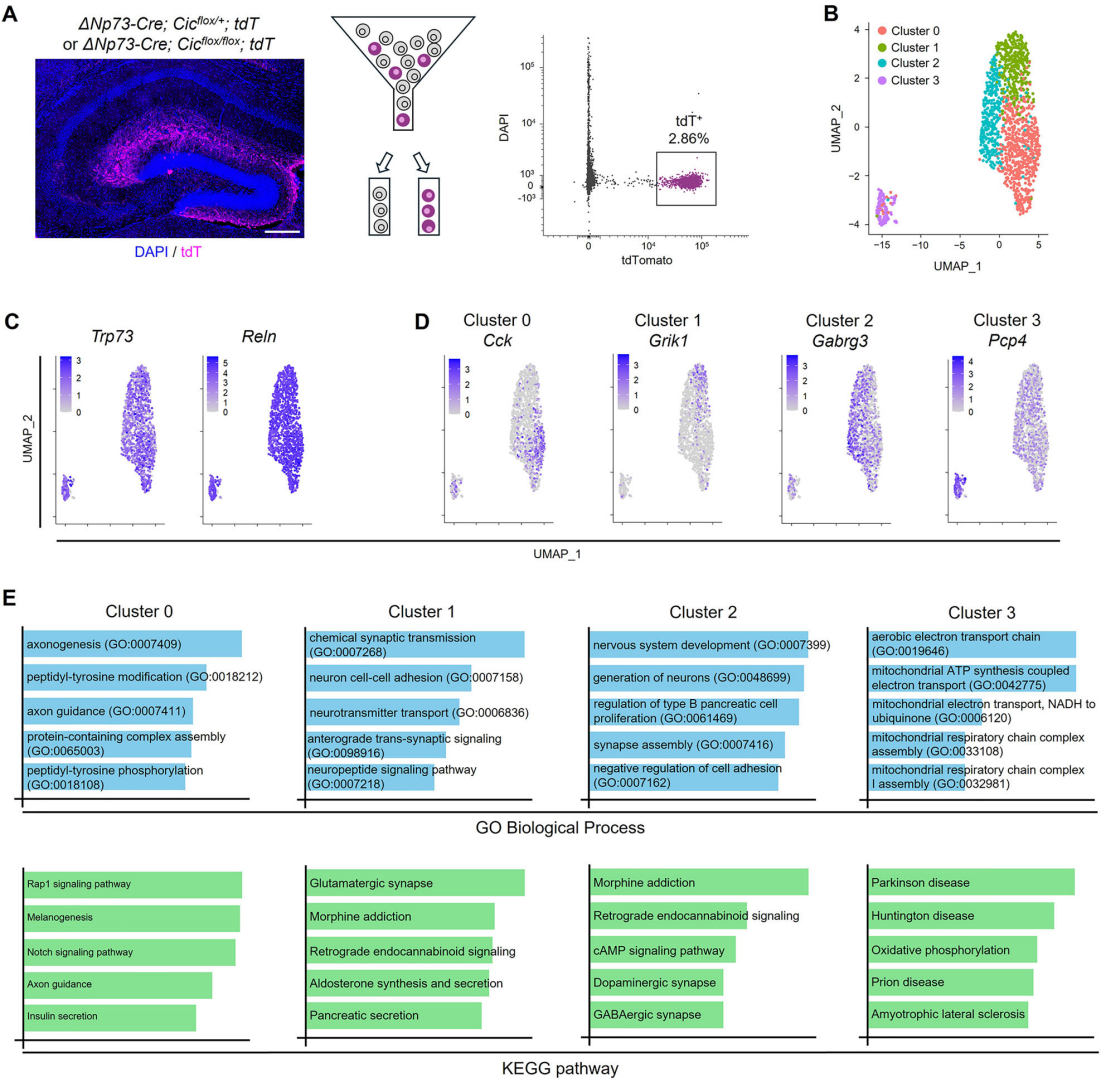
Single-cell transcriptomics reveals distinct subtypes of *ΔNp73*-lineage Cajal-Retzius cells in the postnatal hippocampus. **A** *Left:* A representative confocal image shows the hippocampus of a *ΔNp73-Cre; Cic*^*flox/+*^*; tdT* mice at postnatal day 14. tdT expression at this age is limited to hippocampal CR cells. *Middle:* The hippocampus was dissected for cell preparation and fluorescence activated cell sorting. *Right:* A representative flow cytometry dot plot shows that about 3% of the cell population were tdT-expressing cells. DAPI was used to discriminate live versus dead cells. **B** 2D uniform manifold approximation and projection (UMAP) plot reveals four distinct clusters of CR cells. **C** UMAP plots showing *Trp73* and *Reln* expression in all clusters. **D** UMAP plots showing enriched expression of selective marker genes in each of the clusters. **E** Gene ontology and functional pathway analyses of maker genes (*adjP* < 0.05) of each cluster. Only the top five most enriched gene ontology terms and pathways are shown based on their significance (*q*-value). Terms and pathways with the lowest *q*-value are at the top.

In the embryonic brain, CR cells arise from four distinct progenitor domains, including the ventral pallium, the septum, the thalamic eminence, and the cortical hem [6, 43, 44], but whether additional heterogeneity exists within each ontogenetically defined subtype is unknown. Hippocampal CR cells originate from the cortical hem and are targeted by the *ΔNp73-Cre* [10, 11]. To gain insight into the transcriptional identity of hippocampal CR cells, we performed single-cell RNA-sequencing (scRNA-seq) on CR cells isolated from the *ΔNp73-Cre; Cic*^*flox/+*^*; tdT* (conditional heterozygous) and the *ΔNp73-Cre; Cic*^*flox/flox*^*; tdT* (conditional knockout) mouse hippocampus at P14. We opted to use the conditional heterozygous mice as controls as they did not show any CR cell death or behavior deficits (Figs. 1–2) and allowed us to pool sufficient animals from the same litters on the same day for the single-cell study. We chose P14 because hippocampal CR cells undergo rapid elimination at this stage [13, 14], and we had observed reduced TUNEL^+^ apoptotic cells at this stage following *Cic* deletion (Fig. S2) or *Bcl2* overexpression (Fig. S5B). Hippocampi from P14 conditional heterozygous or conditional knockout mice were dissected, and *tdT*-expressing CR cells (~ 3% of total cells) were isolated via fluorescence-activated cell sorting followed by single-cell sequencing (Fig. 4A). Analysis of the single-cell data revealed four clusters (Fig. 4B). *Trp73* and *Reln*, selective markers of CR cells, were highly expressed in all four clusters, validating our CR cell enrichment approach and their *ΔNp73*-lineage CR cell identity (Fig. 4C). Marker gene analysis of each cluster showed that the neuropeptide gene *Cck* was highly selective for cluster 0 (Fig. 4D). Gene ontology and functional pathway analysis further showed that cluster 0 was highly enriched for genes related to axon development (Fig. 4E and Table S1). Interestingly, cluster 1 cells selectively expressed the glutamate receptor *Grik1* and these cells were enriched for genes related to “Glutamatergic synapse”, while cluster 2 cells expressed high levels of the GABA_A_ receptor *Gabrg3* and were enriched for genes related to “GABAergic synapse” (Fig. 4D, E). Cluster 3 was a distinct cluster segregated away from the other three clusters and cells in this cluster express high levels of the small calmodulin-binding protein *Pcp4*. Marker genes in cluster 3 were highly enriched for terms related to mitochondria function and neurodegenerative diseases, suggesting that this might be a cluster of CR cells undergoing active cell death. Overall, our single-cell transcriptomic analysis identified distinct subsets of CR cells in the early postnatal hippocampus, uncovering previously unappreciated heterogeneity of *ΔNp73*-lineage CR cells.

### *Fgf1* upregulation enhances Cajal-Retzius cell survival in the postnatal hippocampus

**Fig. 5:**
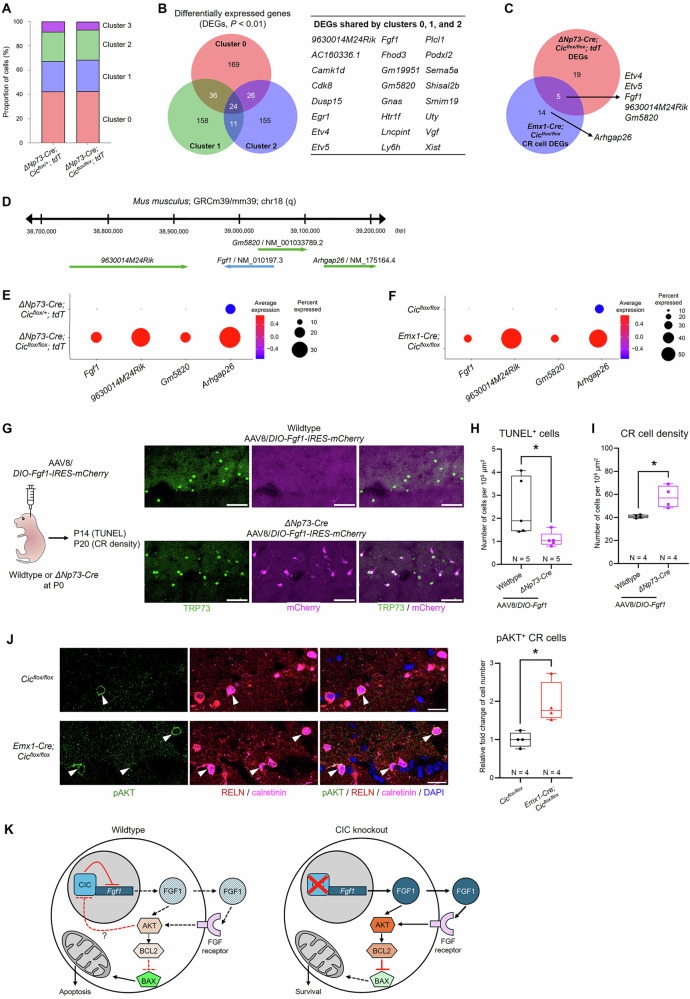
*Fgf1* upregulation enhances Cajal-Retzius cell survival in the postnatal hippocampus. **A** The proportion of cells within each cluster is similar between the *ΔNp73-Cre; Cic*^*flox/+*^*; tdT* and the *ΔNp73-Cre; Cic*^*flox/flox*^*; tdT* samples. **B** Venn diagram of differentially expressed genes (DEGs) in clusters 0, 1 and 2 between the *ΔNp73-Cre; Cic*^*flox/+*^*; tdT* and the *ΔNp73-Cre; Cic*^*flox/flox*^*; tdT* datasets. The 24 genes found in the overlap of all three clusters are shown on the right. **C** Venn diagram showing the overlap of DEGs between the *ΔNp73-Cre; Cic*^*flox/flox*^*; tdT* dataset and the *Emx1-Cre; Cic*^*flox/flox*^ dataset. **D** Illustration of the *Fgf1* locus in the mouse genome shows *Fgf1* and three other adjacent transcripts. **E, F** Dot plots showing increased expression of *Fgf1*, *9630014M24Rik*, *Gm5820*, and *Arhgap26* in the *ΔNp73-Cre; Cic*^*flox/flox*^*; tdT* dataset (**E**) and in the *Emx1-Cre; Cic*^*flox/flox*^ dataset **F**. These four genes are located in or adjacent to the *Fgf1* locus in mice. **G** *Left*: Overview of the intracerebroventricular injection and experimental timeline. *Middle*: Representative images of TRP73^+^ CR cells along the hippocampal fissure from postnatal day (P) 20 wildtype and *ΔNp73-Cre* mice injected with the AAV. Scale bars = 50 µm. **H** Quantification of TUNEL-positive cell density along the hippocampal fissure at P14. Statistical analysis was performed using a nested *t*-test, with 3–4 brain sections analyzed per animal and sections treated as nested observations. Each data point represents one animal. **I** Quantification of CR cell density along the hippocampal fissure at P20. Each data point represents one animal, with values averaged from three coronal hippocampal sections. Statistical analysis was performed using Welch’s *t*-test. **J** *Left*: Representative images of phosphorylated AKT (pAKT)–positive CR cells (arrowheads) along the hippocampal fissure in P11 *Cic*^*flox/flox*^ and *Emx1-Cre; Cic*^*flox/flox*^ mice. Scale bars = 20 µm. *Right*: Quantification of the relative fold change in pAKT⁺ CR cell number, normalized to littermate controls. Each data point represents one animal, with values averaged from three coronal hippocampal sections. Statistical analysis was performed using Welch’s *t*-test. Data are presented as box-and-whisker plots with all data points shown. *, *p* < 0.05. **K** A working model that outlines the key regulators of hippocampal CR cell apoptosis versus survival in the presence and absence of CIC.

After quality control, we recovered 1013 cells from the *ΔNp73-Cre; Cic*^*flox/+*^*; tdT* control and 389 cells from the *ΔNp73-Cre; Cic*^*flox/flox*^*; tdT* knockout scRNA-seq datasets. The lower recovery in the knockout samples may reflect increased vulnerability of these cells to dissociation and sorting. Nonetheless, analysis of the two datasets did not reveal any clusters unique to the knockout mice, and the two groups of mice shared similar proportions of each cell cluster (Fig. 5A). To identify the signaling pathway that regulates developmental CR cell death, we compared the differentially expressed genes (DEGs) of clusters 0, 1, and 2 between the two datasets (Fig. 5B). We excluded cluster 3 from this analysis due to the possibility that the cluster is undergoing active cell death which may preclude the identification of upstream signaling pathways prior to overt cell death. We found 24 DEGs shared by all three clusters, which include known CIC target genes *Etv4*, *Etv5*, and *Vgf* [45, 46], validating our single-cell approach and the conditional heterozygous as an appropriate control (Fig. 5B).

Because abnormal CR cell persistence was observed in both *Emx1-Cre; Cic*^*flox/flox*^ and *ΔNp73-Cre; Cic*^*flox/flox*^ mice (Fig. 1), we next leveraged our published single-nucleus RNA-seq dataset from P19 *Cic*^*flox/flox*^ control and *Emx1-Cre; Cic*^*flox/flox*^ knockout hippocampi [47]. Within this dataset, we bioinformatically isolated the CR cell cluster based on *Trp73* and *Reln* expression (Fig. S7). After quality control, we recovered 142 CR cells among 17,749 total control cells and 195 CR cells among 16,871 total knockout cells. DEG analysis between control and knockout CR cells identified 19 genes, five of which—*Etv4, Etv5, Fgf1, 9630014M24Rik, and Gm5820*—overlapped with the *ΔNp73-Cre* dataset (Fig. 5C). Intriguingly, *Fgf1*, *9630014M24Rik*, and *Gm5820* were consistently upregulated in both knockout models and are located in close proximity on the mouse genome (Fig. 5D–F**)**. Moreover, another gene in this locus, *Arhgap26*, was upregulated in clusters 0 and 1 of the *ΔNp73-Cre* dataset as well as in the *Emx1-Cre* knockout dataset (Fig. 5C–F). Together, these findings suggest that CIC loss in hippocampal CR cells de-represses the *Fgf1* genomic locus, pointing to FGF1 signaling as a candidate driver of their enhanced survival.

FGF1 is a neurotrophic factor that promotes neuronal cell survival in vivo and in vitro [48–53]. We next tested whether FGF1 overexpression could promote abnormal CR cell survival in the postnatal hippocampus. To this end, we delivered AAV carrying a Cre-dependent *Fgf1* construct (AAV8/*DIO-mFgf1-IRES-mCherry*) via intracerebroventricular injection into P0 *ΔNp73-Cre* or wildtype pups (Fig. 5G). TUNEL staining at P14 revealed fewer apoptotic cells in *ΔNp73-Cre* mice injected with AAV8/*DIO-mFgf1* (Fig. 5H). By P20, these mice displayed a significant increase in surviving CR cells (Fig. 5I), indicating that *Fgf1* upregulation is sufficient to enhance CR cell survival in the postnatal hippocampus. The pro-survival actions of FGF1 on neurons are thought to be mediated by either the MAPK pathway or the PI3K/AKT/mTOR pathway [54, 55], which inhibits apoptosis via BAX downregulation or BCL2 upregulation [48, 56, 57]. We first confirmed that the FGF1 receptor *Fgfr1* was expressed by a subset of CR cells across clusters 1–3 in the *ΔNp73-Cre* scRNA-seq dataset (Fig. S8). We then examined pathway activation by quantifying CR cells positive for phosphorylated ERK1/2 (pERK1/2, MAPK signaling) or phosphorylated AKT (pAKT, PI3K/AKT/mTOR signaling) in control and *Emx1-Cre; Cic*^*flox/flox*^ knockout mice at P11, prior to the onset of rapid CR cell death [13, 14]. Notably, knockout mice showed more CR cells immunoreactive for pAKT (Fig. 5J) and fewer positive for pERK1/2 (Fig. S8B), indicating that FGF1 enhances CR cell survival primarily through the PI3K/AKT/mTOR pathway rather than MAPK signaling (Fig. 5K).

## Discussion

The mechanisms of CR cell death in the hippocampus remain largely elusive but were once considered largely non-apoptotic [12, 58]. However, recent studies demonstrate that at least some hippocampal CR cells undergo apoptosis-dependent cell death [20]. Nonetheless, upstream regulators of CR cell apoptosis remain largely unexplored. In the present study, by combining *Cic* knockout mouse models, single-cell transcriptomics, and viral-mediated gene delivery to CR cells, we identified the FGF1–BCL2 axis acting downstream of CIC to regulate CR cell apoptosis in the postnatal hippocampus.

Our data show that *Cic* loss from CR cells results in a similar increase in CR cell density as seen with *Bax* knockout mice and mouse models of sustained PI3K-AKT-mTOR pathway activation. However, we did not observe behavioral abnormalities or altered seizure susceptibility in the CR-cell-specific *Cic* knockout mice. One possibility is that, compared to CIC, BAX and the PI3K-AKT-mTOR pathway may have additional roles in hippocampal CR cells beyond apoptosis regulation. For example, BAX plays a role in mitochondria metabolism and calcium homeostasis at the endoplasmic reticulum [59], and the PI3K-AKT-mTOR pathway regulates glycolytic metabolism and autophagy [60, 61]. It is possible that the behavioral deficits and/or altered seizure threshold in the *Bax* knockout mice and mouse models of PI3K-AKT-mTOR activation are due to the roles of these factors beyond apoptosis. Another possibility is that CIC is required in abnormally persisting CR cells to alter cognitive function and seizure susceptibility. How CIC may do so remains to be investigated, but the loss of CIC may impair the activity of abnormally surviving CR cells. Previous studies have highlighted a role of CIC in neuronal maturation [28, 34]. Therefore, CIC deletion may impair morphological and/or functional maturation of CR cells, leading to compromised activity and/or connectivity of these cells. However, it is also worth noting that CIC deletion does not completely abolish CR cell activity, for if that is the case, then one would expect behavioral deficits reminiscent of those seen in the CR cell-specific *Vglut2* knockout mice [18]. Since we do not detect any noticeable behavioral deficits in the CR cell-specific *Cic* knockout mice, we hereby propose that learning deficits and seizures seen in individuals with the *CIC*-related neurodevelopmental disorder are unlikely to be caused by CIC dysfunction in CR cells.

CR cells are recognized to be heterogeneous as defined by their diverse ontogenetic origins. In rodents, CR cells originate from the cortical hem, the ventral pallium, the septum, and the thalamic eminence [36, 39, 62–64]. A previous study described substantial transcriptional diversity among CR cells in the E18 dorsal and medial pallium using a down-sampling–deconvolution framework that resolved eight CR cell clusters [65]. In contrast, our P14 hippocampal CR cell scRNA-seq dataset revealed four clusters with little overlap in marker genes. This discrepancy is expected, given the differences in developmental stage (embryonic vs. postnatal), anatomical context (whole forebrain vs. hippocampus), and analytical approach (down-sampling/deconvolution vs. standard single-cell pipeline). Hippocampal CR cells arise from the cortical hem and belong to the *ΔNp73*-lineage. Our single-cell transcriptomics study revealed that, even within an ontogenetically homogeneous group of *ΔNp73*-lineage hippocampal CR cells, there is recognizable heterogeneity defined by their transcriptional signatures. Our data uncover unique developmental states and functional subgroups of hippocampal CR cells. Cells in cluster 0 may be involved in “axonogenesis” and “axon guidance” as indicated by gene set enrichment analysis. Hippocampal CR cells form elaborate axon collaterals. Based on their projection patterns, hippocampal CR cells can be grouped into three subtypes [12]. Whether cluster 0 CR cells in our dataset fall into a specific projection pattern subgroup remains to be investigated. Nonetheless, our data provide a useful resource to further dissect genes and pathways important for axon development of hippocampal CR cells. Our data also reveal potential functional subtypes of hippocampal CR cells in the local circuit. Hippocampal CR cells receive GABAergic inputs from neurogliaform interneurons in the SLM and interneurons in the OLM [16, 66], and these spontaneous synaptic inputs are completely abolished by GABA_A_ receptor antagonists [66, 67]. Our identification of cluster 2 CR cells expressing high levels of *Gabrg3*, which encodes the γ subunit of the GABA_A_ receptor, suggests that different subsets of hippocampal CR cells may have varying levels of the γ3 subunit, which could affect receptor localization and functions [67–69]. Although hippocampal CR cells receive almost exclusively GABAergic inputs, they also express functional ionotropic glutamate receptors [67]. Our data indicate that the kainate receptor subunit *Grik1* is selectively expressed in cluster 1 cells, shedding light on the molecular composition of the putative ionotropic glutamate receptor complexes.

In the absence of CIC, *Fgf1* and several immediate neighboring transcripts are upregulated in hippocampal CR cells. This strongly indicates the involvement of an enhancer element that regulates these transcripts. As a transcriptional repressor protein, CIC is recruited to promoters, enhancers, and distal intergenic regions [45, 70, 71]. However, whether CIC regulates the *Fgf1* locus directly or indirectly remains to be investigated. Our data contrast with the prevailing model that CIC acts exclusively downstream of the growth factor and RAS/MAPK pathway [23–25]. Instead, we find that CIC acts upstream of the growth factor signaling pathway by repressing *Fgf1* expression, highlighting a context-dependent role of CIC in growth factor signaling. The inter-regulation of CIC and FGF signaling may constitute a positive feed-forward loop, whereby FGF signaling downregulates CIC function via the RAS/MAPK pathway, and reduced CIC function further drives upregulation of FGF1. Whether and how this putative loop plays a role in normal brain development remains to be seen, but FGF8 signaling is necessary and sufficient to specify CR cells originating from the rostral septum [72]. Effectors of FGF8 signaling, *Etv1*, *Etv4*, and *Etv5*, are also direct CIC target genes [25, 73, 74]. This raises the intriguing possibility that CIC might also regulate the expression of FGF signaling effectors to specify rostral versus caudal CR cells in the embryonic brain.

FGF1 may act intracellularly or be secreted via nonclassical release [48, 52, 75–78]. In the CR-cell-specific *Cic* knockout mice, whether the upregulated FGF1 acts intracellularly or extracellularly, via autocrine or paracrine pathways, remains to be determined. Our findings suggest that the pro-survival effect of FGF1 on CR cells is mediated through the PI3K/AKT/mTOR pathway, which inhibits apoptosis in part by upregulating BCL2, as previously proposed [56, 79, 80]. Based on these results, we propose an integrated model for the regulation of apoptosis of CR cells in the postnatal hippocampus (Fig. 5K). In this model, CIC normally represses *Fgf1* expression, thereby restraining PI3K/AKT/mTOR activity and promoting apoptosis through BAX upregulation and/or BCL2 downregulation. In the absence of CIC, FGF1 signaling is de-repressed, leading to enhanced AKT phosphorylation, increased BCL2 levels, and prolonged CR cell survival. Thus, CIC functions as a critical “sensor” that tunes the threshold for pro-survival signaling in hippocampal CR cells. However, the upstream sources and nature of the survival cues, and how they regulate CIC activity, remain open questions.

Taken together, our scRNA-seq data offer a rich resource to further investigate key players in synaptic transmission and define the functional subtypes of hippocampal CR cells. Our study reveals a previously unrecognized role of CIC upstream of FGF1 and provides deeper insights into the mechanisms that regulate CR cell apoptosis in the postnatal hippocampus.

## Methods

### Animals

Generation of the *Cic*^*flox*^ mice has been previously described [28]. The *Cic*^*flox*^ mice are also available from The Jackson Laboratory (stock number: 030555). Wildtype C57BL/6 J mice, *Emx1-Cre* mice [B6.129S2-*Emx1*^*tm1(cre)Krj*^/J, stock number: 005628], and *LSL-tdTomato* [B6.Cg-*Gt(ROSA)26Sor*^*tm9(CAG-tdTomato)Hze*^*/J*, stock number: 007909] [81] mice were obtained from The Jackson Laboratory. *ΔNp73-Cre* mice [39] were a generous gift from Dr. A. Pierani (Université de Paris, Paris, France). Mice were group-housed in a 12-hour light – 12-hour dark cycle, with all experiments performed during the light period. Both male and female mice were used for experiments. Detailed information regarding the number of animals used in each experiment is provided in each figure. Ages are indicated where applicable. Animals were not intentionally randomized into experimental groups. However, experiments were generally conducted in the order of animal identification numbers (from smallest to largest), which introduced a quasi-random sequence in the allocation and processing of samples. Data collection and quantification were performed by investigators blinded to genotype and treatment conditions. All animal procedures were performed in accordance with the animal care and use committee regulations of the University of Alberta.

### Adeno-associated viruses and neonatal intracerebroventricular injection

*pAAV[FLEXon]-SYN1* > *LL:rev(mFgf1[NM_010197.3]:IRES:mCherry):rev(LL):WPRE* and *pAAV[FLEXon]-EF1A* > *LL:rev(V5/3xGS/mBcl2[NM_009741.5]):rev(LL):WPRE* were designed and produced by VectorBuilder. *pAAV-EF1α-double floxed-hChR2(H134R)-mCherry-WPRE-HGHpA* was a gift from Karl Deisseroth [Stanford University, Viral Prep #20297-AAV8, Addgene (http://n2t.net/addgene:20297); RRID:Addgene_20297].

shRNAs targeting mouse *Bcl2* were designed using the Genetic Perturbation Platform (GPP) from the Broad Institute. To generate the miR-30E–based Cre-dependent AAV/shRNA constructs (pAAV/*EF1α-FLEXon-mCherry-miR30E-shRNA*), a DNA fragment flanked by *KpnI* (5′) and *HindIII* (3′)—containing, in order: lox2272, mCherry, 5′ miR-30E, shRNA, 3′ miR-30E, and loxP—was synthesized by Twist Bioscience. This fragment was digested with *KpnI* and *HindIII* and ligated into *KpnI/HindIII*-digested *pAAV[FLEXon]-EF1A* > *LL:rev(V5/3xGS/mBcl2[NM_009741.5]):rev(LL):WPRE*. For the U6-based Cre-dependent AAV/shRNA constructs (pAAV/*CreON-shRNA*), a DNA fragment flanked by *HpaI* (5′) and *SpeI* (3′)—containing the shRNA and a terminator sequence—was synthesized by Twist Bioscience. This fragment was digested with *HpaI* and *SpeI* and ligated into *HpaI/SpeI*-digested pAAV-R-CreON shRNA[Control] [82], a gift from Eun Mi Hwang; Addgene plasmid #180775, RRID:Addgene_180775. All DNA constructs were validated by nanopore full-length plasmid sequencing. AAV plasmids were amplified in NEB Stable *E. coli* (C3040H; New England Biolabs, catalog #C3040H).

Viruses were aliquoted and stored at −80 °C until use. Thawed viruses were kept at 4 °C and used within a week. Neonatal intracerebroventricular injections of viruses were performed as previously described [14]. Briefly, newborn pups within 6-hours of birth were anesthetized via hypothermia. The cranial surface was disinfected, and 2 μL of AAV at 1.0–5.0×10^12^ genome copy (GC)/μL was injected into each ventricle using a gas-tight syringe with a 32-gauge needle. After injection, the cranial surface was disinfected, and the pup was placed on a heating pad until mobility and cardiac output were restored. Pups were returned to the home cage and monitored daily for one week.

### In vitro testing of Bcl2 shRNA

HEK293T cells were purchased from ATCC, tested for mycoplasma contamination prior to expansion, and subsequently cryopreserved. Cells cultured in 6-well plates were transfected with 1000 ng total plasmid DNA using Lipofectamine 3000 (catalog #L3000008, Thermo Fisher Scientific). Each transfection included 500 ng of pAAV/shRNA plasmid (scramble or *Bcl2* shRNA, miR-30E- or U6-based) and 500 ng of an overexpression plasmid (empty vector control or *pAAV[FLEXon]-EF1A* > *LL:rev(V5/3xGS/mBcl2[NM_009741.5]):rev(LL):WPRE*). At 72-hour post-transfection, cells were lysed, and protein was isolated for immunoblotting as previously described [33]. Primary antibodies used for immunoblotting were rabbit anti-BCL2 (1:2000; Abcam, ab182858; RRID:AB_2715467) and mouse anti-GAPDH (1:10,000; Advanced ImmunoChemical, 2-RGM2; RRID:AB_2721282).

### Tissue preparation and immunofluorescence studies

Mice were euthanized and transcardially perfused with PBS and 4% paraformaldehyde (PFA) diluted in PBS. Mouse brains were removed and fixed in 4% PFA overnight at 4 °C, followed by incubation at 4 °C in 15% and 30% sucrose solution for 24 hours each. Brains were then cut coronally using a brain matrix, cryo-embedded into Optimal Cutting Temperature compound (catalog #4585, Fisher HealthCare), and subsequently frozen at −80 °C. Brain sections were cut into 40 µm-thick sections using a Leica CM1520 cryostat (Leica Microsystems Inc., Buffalo Grove, IL) before immunostaining. For phosphorylated AKT immunostaining, tissues were post-fixed by sequentially incubating at room temperature for 5 minutes each in 50%, 75%, 95%, and 100% ethanol, followed by the reverse gradient (100%, 95%, 75%, and 50%), and finally rinsed in PBS. Immunofluorescence staining was performed as previously described [47, 83]. The following primary antibodies were used for immunofluorescence staining: goat anti-tdTomato/mCherry (1:500; AB8181-200, SICGEN; RRID:AB_2722750); rabbit anti-TRP73 (1:500; ab40658, Abcam; RRID:AB_776999); mouse anti-RELN (1:500; MAB5364, MilliporeSigma; RRID:AB_1293544); mouse anti-RELN (1:500; ab78540, Abcam; RRID:AB_1603148); rabbit anti-BCL2 (1:500; ab182858, Abcam; RRID:AB_2715467); rabbit anti-CIC [1:500; Huda Zoghbi Lab; RRID:AB_2721281]; mouse anti-V5; R960-25, Invitrogen; RRID:AB_2556564); rabbit anti-phospho-AKT (Ser473, D9E) [1:200; 4060, Cell Signaling Technologies; RRID:AB_2315049]; rabbit anti-phospho-ERK1/2 [1:200; 9101S, Cell Signaling Technology; RRID:AB_331646. The secondary antibodies used were as follows: donkey anti-goat Alexa Fluor 555 (1:1000; A21432, Thermo Fisher Scientific; RRID:AB_2535853); donkey anti-rabbit Alexa Fluor 488 (1:1000; A21206, Thermo Fisher Scientific; RRID:AB_2535792); and donkey anti-mouse Alexa Fluor 647 (1:1000; A31571, Thermo Fisher Scientific; RRID:AB_162542).

### 5-Ethynyl-2′-deoxyuridine (EdU) Injections and detection

EdU (50 mg/kg) was administered intraperitoneally to P5 mouse pups twice, with a 2-hour interval between injections. Brain tissues were collected 2 hours after the second injection and prepared as described above. EdU detection in combination with co-immunofluorescence was performed using the Click-iT EdU Cell Proliferation Kit for Imaging (Alexa Fluor 555 dye; catalog #C10338, Thermo Fisher Scientific) according to the manufacturer’s instructions [34]. EdU detection was carried out after secondary antibody incubation, with samples protected from light, and sections were counterstained with DAPI before mounting.

### TUNEL staining

TUNEL staining was performed using the Click-iT Plus TUNEL Assay Kit for In Situ Apoptosis Detection (Alexa Fluor 488; catalog #C10617, Thermo Fisher Scientific) according to the manufacturer’s instructions with modifications. Coronal brain sections (10 µm, PFA-fixed) were mounted onto Superfrost Plus slides (catalog #12-550-5, Fisher Scientific). Sections underwent ethanol fixation/clearing by sequential immersion (5 min each at room temperature) in 50%, 75%, 95%, and 100% ethanol, followed by the reverse gradient, and were rinsed in PBS. Post-fixation was performed with warm (35–42 °C) 4% PFA for 15 min at room temperature, followed by PBS washes, and tissue boundaries were circled using a hydrophobic barrier pen. Sections were permeabilized with proteinase K for 15 min at 37 °C, post-fixed again in 4% PFA for 5 min, and rinsed with PBS and deionized water. The TdT reaction was carried out by pre-incubating sections with TdT reaction buffer for 10 min at 37 °C, followed by incubation with the TdT reaction mixture for 1 h at 37 °C. Slides were rinsed in deionized water and washed with 3% BSA/0.1% Triton X-100 in PBS. The Click-iT Plus reaction cocktail was then applied for 30 min at 37 °C in the dark, followed by washes in 3% BSA/PBS and PBS. All incubation steps were performed in a humidified chamber in the dark, with slides covered by glass coverslips to reduce evaporation; coverslips were removed and replaced between steps as required. Subsequent immunofluorescence staining was performed as described above, including citrate antigen retrieval and overnight primary antibody incubation at 4 °C.

### Confocal microscopy and image analysis

Immunofluorescence images were acquired using a Zeiss LSM 700 laser-scanning confocal microscope. For adult mice (>7 weeks old), three coronal sections per animal spanning the hippocampus at Bregma –1.46 mm, –1.94 mm, and –2.46 mm were selected for imaging. For younger mice, anatomically comparable sections were chosen. Tiled and z-stacked images were collected for each animal. To quantify CR cell density, one hemisphere of each section was imaged at 20× magnification with a 10 µm Z-stack. Maximum intensity projections were used for quantification. The length of the hippocampal fissure and the area surrounding the fissure (defined as 60 µm above and below the fissure along its length) were measured using Fiji (ImageJ). CR cells expressing the relevant markers were manually counted within this region across three sections per animal. Cell density was normalized to both the length and area of the hippocampal fissure, and in some cases further normalized to littermate controls subjected to the same treatment or analysis to minimize variability between litters and experimental procedures. To detect BCL2, pERK1/2, or pAKT immunoreactivity in CR cells, single-tile images were acquired at 40× magnification with a 15 µm Z-stack in three regions along the hippocampal fissure per section (nine images per animal in total). Both Z-stack images and maximum intensity projections were used for quantification, and results were reported as the total number of marker-positive CR cells across the nine images per animal. All image acquisition and cell counting were performed by investigators blinded to genotype and treatment conditions.

### Single-cell RNA Sequencing and data analysis

Four *ΔNp73-Cre; Cic*^*flox/+*^*; tdT* and four *ΔNp73-Cre; Cic*^*flox/flox*^*; tdT* mice (littermates) at P14 were euthanized with sodium pentobarbital and perfused with artificial cerebrospinal fluid (aCSF; 87 mM NaCl; 2.5 mM KCl; 1.25 mM NaH_2_PO_4_; 26 mM NaHCO_3_; 75 mM sucrose; 20 mM glucose; 2 mM CaCl_2_; 2 mM MgSO_4_) equilibrated in carbogen (95% O_2_, 5% CO_2_) via a gas stone. The brain was promptly removed and kept in aCSF as the hippocampus was dissected. Dissected tissue was kept submerged in aCSF saturated with carbogen on ice. The cell suspension was prepared using the Papain Dissociation System (catalog # LK003150, Worthingon Biochemical Corporation). The resulting cell pellet was resuspended in oxygenated aCSF and DAPI was added for live/dead cell discrimination. Cells were then sorted using a SONY MA900 cell sorter. Sorted DAPI^−^ tdT^+^ cells suspended in aCSF were used to generate the gel-beads cell emulsion by the 10x Chromium Controller (PN-1000202). Gene expression libraries were prepared using the Chromium Next GEM Single Cell 3’ Gene Expression v3.1 (Dual Index) (catalog #PN-1000269, 10x Genomics) following the manufacturer’s protocol. Sample libraries were sequenced in Illumina NovaSeq 6000 (PE150) to an average depth of ~ 50,000 reads per cell. The resulting FASTQ files were converted to count matrix files using Cell Ranger Count v7.1.0 with Mouse (mm10) 2020-A as the reference genome.

Quality control, dimensionality reduction, and clustering were performed in RStudio (2023.12.0 Build 369) using Seurat (v4.4.0; https://github.com/satijalab/seurat). Briefly, for both heterozygous control and knockout datasets, low-quality nuclei were removed by excluding cells with high or low mitochondrial content (±3 median absolute deviations [MAD] from the median), low gene counts (log10(nFeature_RNA) < median − 3×MAD), high transcript counts (log10(nCount_RNA) > median + 3×MAD), or deviations from the expected nCount/nFeature relationship. Doublets were detected using DoubletFinder with adjustment for homotypic proportions. The two datasets were merged, and transcripts associated with mitochondrial genes (^mt-) and ribosomal/rRNA-related genes (Rpl, Rps, Mrp) were excluded. Each dataset was then independently normalized, and 2000 variable features were selected using the “vst” method. Integration anchors were identified across datasets, and data were integrated to correct for batch effects. Integrated data were scaled, subjected to principal component analysis (PCA, npcs = 30), and clustered using the first 20 PCs (resolution = 0.5). Uniform manifold approximation and projection (UMAP) was used for visualization. Differentially expressed genes (DEGs) between conditions within each cluster were identified using Seurat’s FindMarkers (min.pct = 0.25, log2FC threshold = 0.25). Gene set enrichment analysis was performed using the Enrichr-KG [84] web server (https://maayanlab.cloud/enrichr-kg).

### Behavior tests

One hour prior to each behavior experiment, mice were transported from the housing suite to the testing suite to be habituated with a white noise machine that played throughout all behavior tests. Each behavior test was performed on a separate day. All mouse behaviors were recorded, tracked, and analyzed using EthoVision 17 (Noldus, Wageningen, the Netherlands). Tracking was dependent on the mouse’s center-point as detected by EthoVision 17. Nose- and tail-points were also defined by the software. After testing, the mouse was placed back into its home cage, and the arena was thoroughly cleaned with 70% ethanol prior to testing the next mouse.

#### Open field assay

Mice were placed in a white 40 cm×40 cm arena. Each mouse’s behavior and movement in a 15-minute period were recorded and tracked. The center zone was defined as a 30 cm×30 cm area in the middle of the arena.

#### Elevated plus maze

The elevated plus maze was made of four plastic acrylic arms of equal length (35 cm). Two of the arms were surrounded by 20 cm tall acrylic walls (referred to as the closed arms), while the other two arms were open (referred to as the open arms). Mice were placed in the center of the arena and were allowed to navigate between the arms freely. Each mouse’s behavior and movement in a 10-minute period were recorded and tracked.

#### Novel object recognition

On the first day, mice were habituated in a white 40 cm×40 cm arena for 5 minutes. On the second day, two identical objects (small glass jars) were placed in the center of the arena. Mice were placed in the arena and were allowed to interact with the objects for 10 minutes. On the third day, one of the identical objects was replaced with a novel object (Lego brick build). Mice were placed in the arena and were allowed to interact with the objects for 15 minutes. Interactions on both days were videotaped and tracked. A valid interaction with the object was defined as the mouse’s nose point coming within 1 cm of the object. Quickly running by, climbing, or sitting on the object was considered to be an invalid interaction. The discrimination index was calculated by dividing the difference between the time spent interacting with the novel versus the familiar object by the sum of the time spent interacting with both objects—a positive index reflects preference for the novel objec,t while a negative index reflects preference for the familiar object.

#### Spatial object recognition

On the first day, mice were habituated in a white 40 cm×40 cm arena for 5 minutes with a spatial reference (checkered sheet) appended to one of the walls. On the second day, two identical objects (small plastic ice packs) were placed in the center of the arena. Mice were placed in the arena and allowed to interact with the objects for 10 minutes. On the third day, one of the objects was moved closer to the spatial reference while the other remained in the same position (static). Mice were placed in the arena and were allowed to interact with the objects for 15 minutes. Interactions with the objects on both days were videotaped and tracked. The discrimination index was calculated by dividing the difference between the time spent interacting with the moved and static object by the sum of the time spent interacting with both objects—a positive index reflects preference for the moved object, while a negative index reflects preference for the static object.

#### Fear conditioning

Fear conditioning was conducted using the Ugo Basile 46000 Fear Conditioning System controlled by EthoVision 17. On the first day, mice were trained with two tone-shock pairings with 80 second intervals in-between. A tone (2 kHz) played for 20 seconds, and a foot shock at 0.5 mA was delivered during the last two seconds. On the second day, mice were placed back in the arena. The time spent immobile (freezing) in the arena was tracked to assess context-dependent memory. On the third day, the walls of the arena were covered with checkmark sheets, the electric grid was covered with a plastic tile, and a cup with vanilla essence was placed in the chamber. Mice were placed in the arena and were able to move freely. Two 20-second-long tones played in 80 second intervals without a foot shock. The time spent frozen in response to the tone was tracked to assess cue-dependent memory.

#### Spontaneous Y maze

Mice were placed in the center of a white Y-shaped arena. Each arm was of equal length (35 cm×5 cm). Mice were able to freely move around the arena and were tracked over an 8-minute period. An alternation was defined as the number of times a mouse visited unique arms sequentially without revisiting a previous arm (i.e., traversing from arm A to B to C counted as 1 alternation, whereas arm A to B to A does not count as an alternation). The alternation index is calculated by dividing the number of alternations by the maximum possible alternations, which is calculated by Ethovision 17.

#### Light–dark box

The light–dark box was divided into two sections—a 25 cm × 40 cm open section enclosed by transparent acrylic walls, and a 17.5 cm×40 cm dark section enclosed by black acrylic walls and a black plastic cover. A 5 cm-wide opening divided the light-exposed and dark sections of the arena. Mice were placed in the light-exposed section and were able to freely navigate between the light and dark regions of the arena for 10 minutes.

#### Kainic acid-induced seizures

8-week or older mice were placed in separate cages and intraperitoneally injected with 20 mg/kg body weight of kainic acid. Mice were monitored over a 90-minute period. Their behavior and seizure intensity were monitored every 5 minutes. Mice were scored according to a modified Racine scale: 0 = normal response; 1 = limited or no mobility; 2 = head nodding; 3 = hunched posture/stasis; 4 = rearing with forelimb clonus; 5 = rearing and falling, wild jumping, or loss of posture tone. Mice that reached a Racine score of 4 were considered to have developed seizures. Mice that had a Racine score of 5 and persisted for more than 5 minutes reached the experimental endpoint.

### Statistical analyses

Sample sizes are indicated in the figure legends and were consistent with our previous studies. All experimental data were obtained from at least three animals. Statistical analyses were performed using GraphPad Prism. Detailed information on statistical analysis and sample size is provided in the figures and their legends.

## Supplementary information

Supplemental figures

Original western blots

Supplementary Table S1. Gene set enrichment analysis of Cajal-Retzius (CR) cell clusters.

## Data Availability

All study data are included in the article and supporting information. Raw single-cell RNA-seq reads and processed count matrix has been deposited into Gene Expression Omnibus (GSE309238).

## Code availability

This paper does not include original code.
