# Supplementary material for: Capicua regulates the survival of Cajal-Retzius cells in the postnatal hippocampus
Source: Cell Death Dis. 2025 Dec 22;16(1):898. doi: 10.1038/s41419-025-08206-7 (PMC12722426; doi:10.1038/s41419-025-08206-7)
Supplement: Supplementary file 1 — Supplemental figures [file 41419_2025_8206_MOESM1_ESM.docx]

**Figure S1**


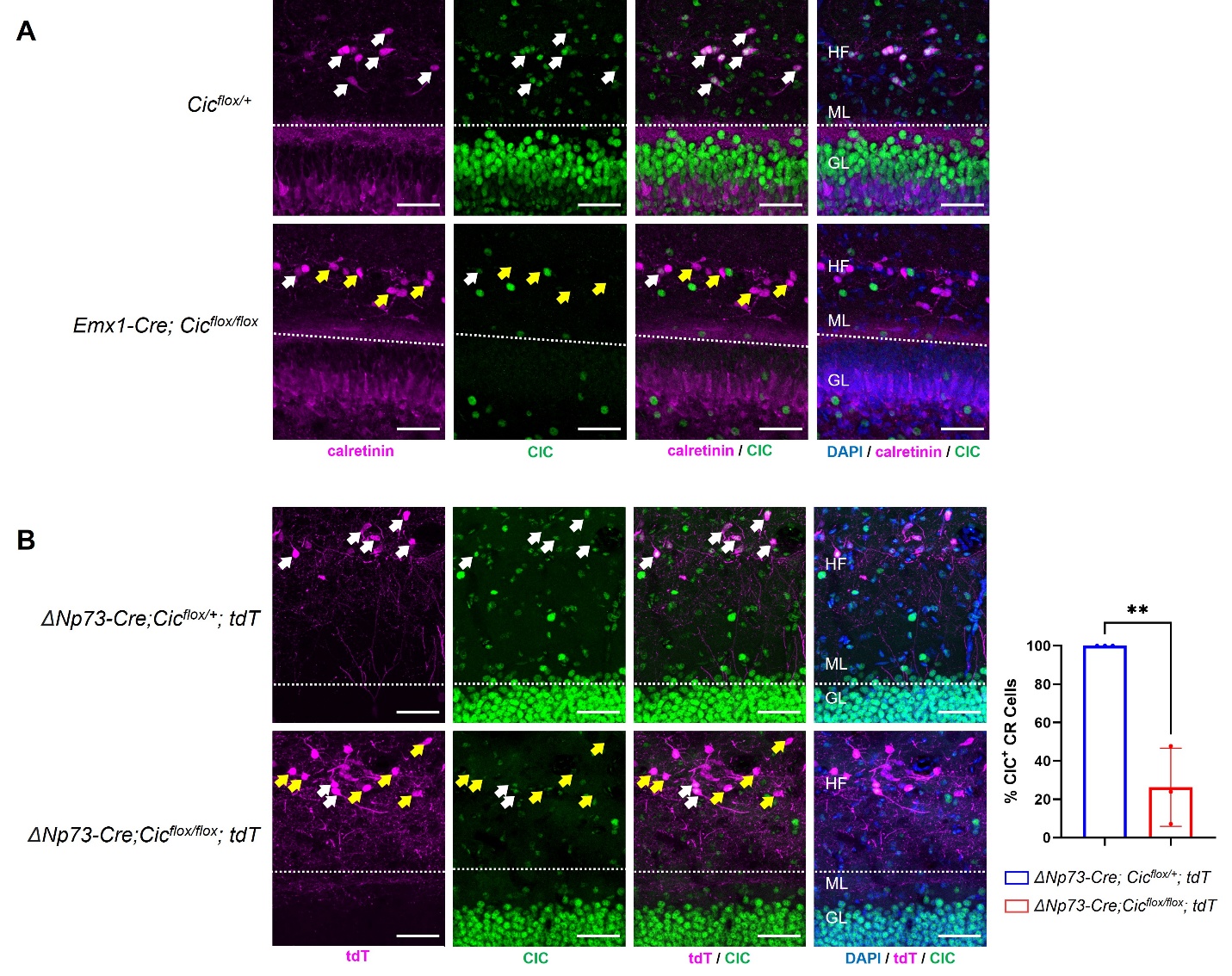


**Figure S1. CIC deletion from hippocampal CR cells in the *Emx1-Cre; Cic^flox/flox^* and the *ΔNp73-Cre; Cic^flox/flox^* mice.** (*A*) Representative immunofluorescence images of CR cells along the hippocampal fissure (HF) in the *Cic^flox/+^* and *Emx1-Cre; Cic^flox/flox^* mice at postnatal day 10. CR cells are identified by calretinin immunoreactivity and their location along the hippocampal fissure. White arrows point to CR cells with CIC expression while yellow arrows point to CIC-depleted CR cells. CIC is deleted from CR cells as well as other forebrain excitatory neurons, including dentate gyrus granule neurons located in the granular layer (GL). (*B*) Representative images of CR cells along the hippocampal fissure in the *ΔNp73-Cre; Cic^flox/+^; tdT* and *ΔNp73-Cre; Cic^flox/flox^; tdT* mice at postnatal day 18. CR cells were identified by their expression of tdT. White arrows point to CR cells with CIC expression while yellow arrows point to CIC-depleted CR cells. Quantification is shown to the right. Data are presented as a scatter plot with error bars representing ± SD. Each data point represents a single animal. Statistical analysis was performed using Welch’s *t*-test. **, *p* < 0.01. Scale bars = 50 µm. ML, molecular layer.

**Figure S2**

**
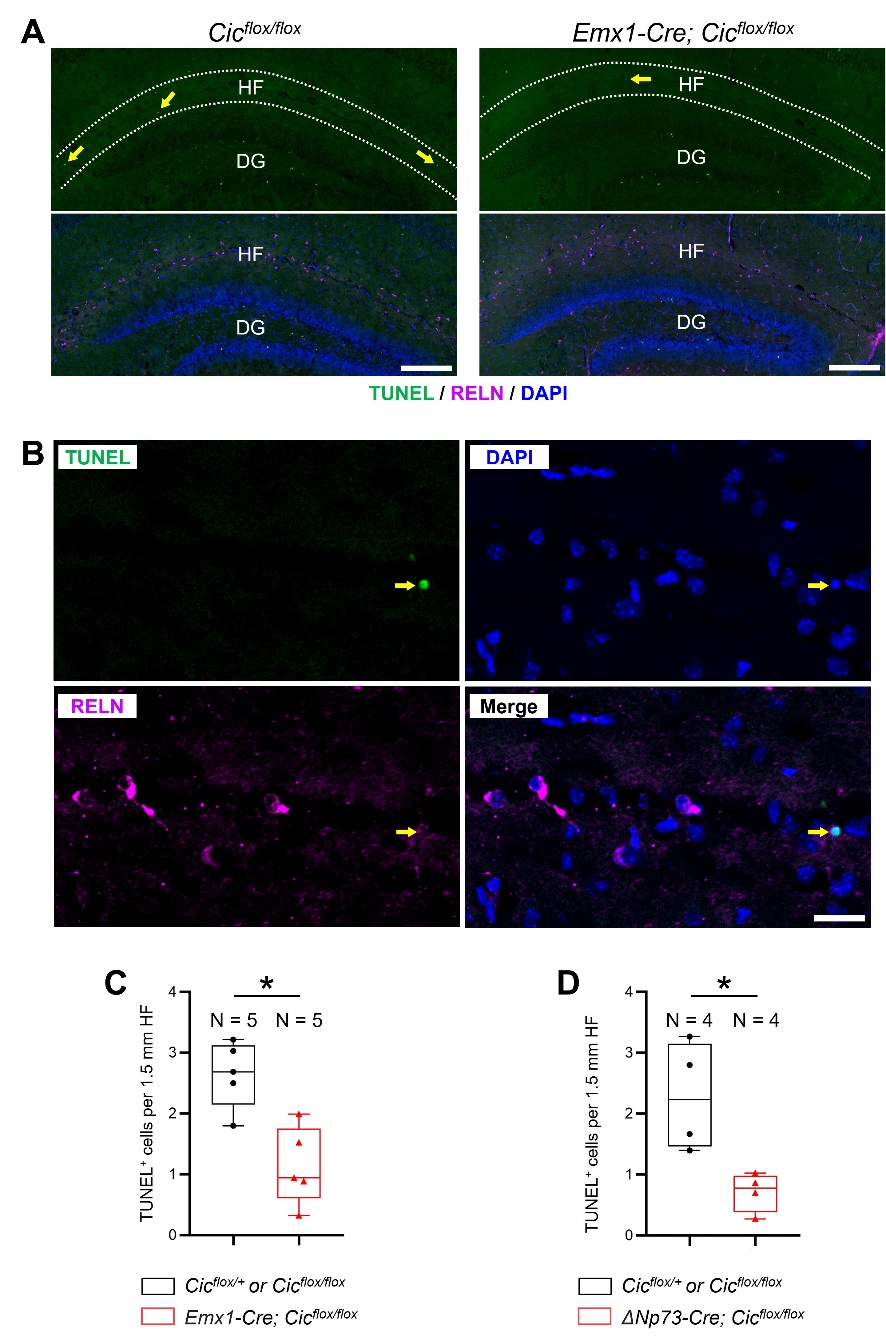
**

**Figure S2. Loss of *Cic* from Cajal-Retzius cells decreases apoptotic cell density along the hippocampal fissure.** (*A*) Representative overview images of the dentate gyrus (DG) at postnatal day (P) 14 from *Cic^flox/flox^* and *Emx1-Cre; Cic^flox/flox^* mice co-stained with RELN and TUNEL. Yellow arrows indicate TUNEL⁺ cells along the hippocampal fissure (HF). Scale bars = 200 µm. (*B*) Representative images of a TUNEL⁺ RELN⁻ CR cell (yellow arrow) along the hippocampal fissure, exhibiting a condensed nucleus visualized by DAPI staining. No TUNEL⁺ signals were detected in RELN⁺ CR cells in either control or knockout hippocampi. Scale bar = 25 µm. (*C* and *D*) Quantification of TUNEL⁺ cell density along the hippocampal fissure at P14 in *Cic^flox/flox^* and *Emx1-Cre; Cic^flox/flox^* mice (*C*) and in *Cic^flox/flox^* and *ΔNp73-Cre; Cic^flox/flox^* mice (*D*). Data are presented as box-and-whisker plots with all data points shown. Each data point represents one animal, with 3–4 brain sections analyzed per animal and treated as nested observations. Statistical analysis was performed using a nested *t*-test. *, *p* < 0.05.

**Figure S3**

**
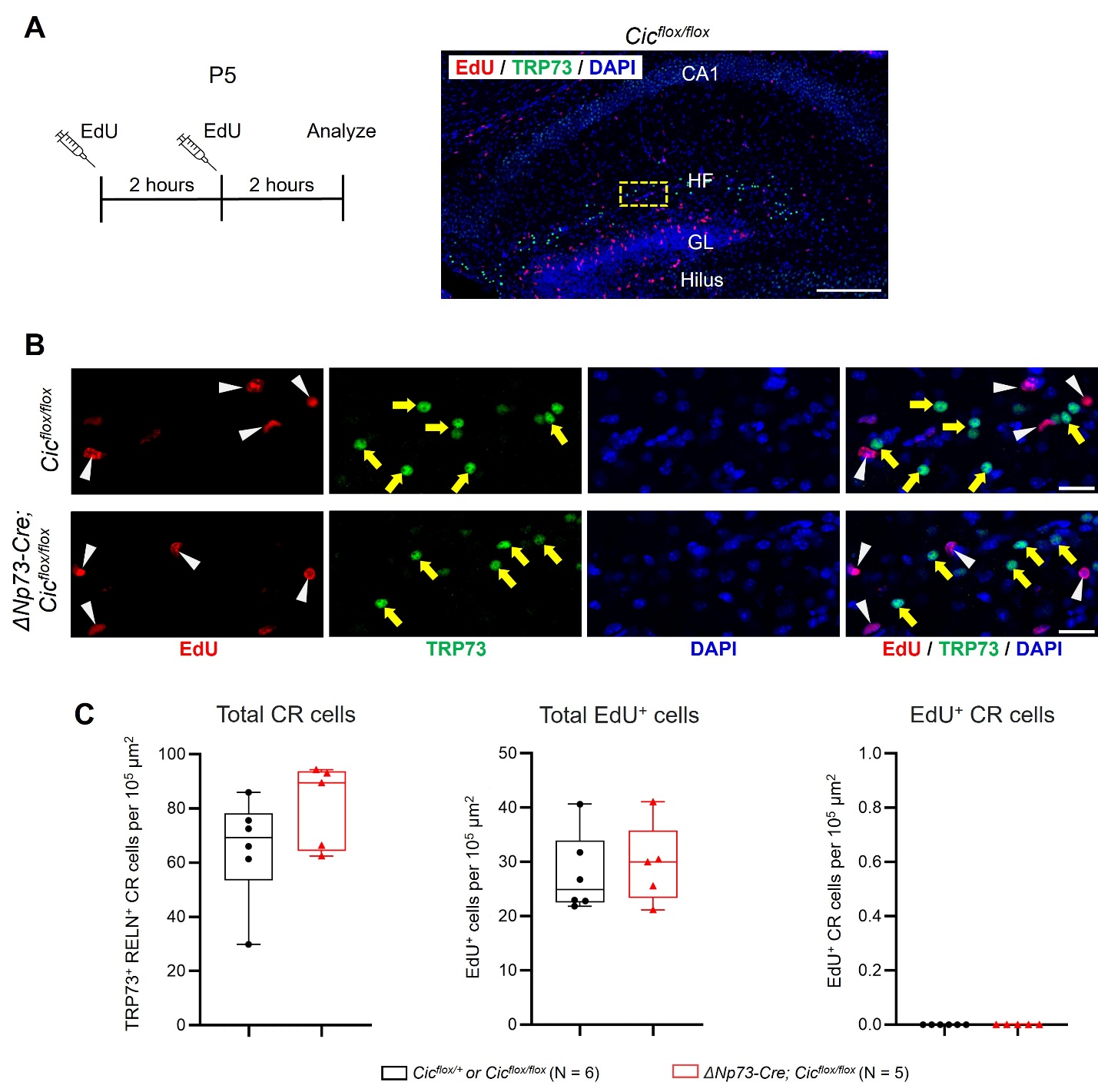
**

**Figure S3. Cajal-Retzius cells are post-mitotic at postnatal day (P) 5.** (*A*) *Left*: Experimental timeline for EdU injection at P5. *Right*: Representative overview coronal hippocampal section from a P5 *Cic^flox/flox^* pup injected with EdU and co-stained with TRP73. CA1, cornu ammonis 1; HF, hippocampal fissure; GL, granule cell layer. Scale bar = 200 µm. (*B*) Representative confocal images of the hippocampal fissure region (boxed in A) from *Cic^flox/flox^* and *ΔNp73-Cre; Cic^flox/flox^* pups injected with EdU at P5. EdU-labeled cells (white arrowheads) do not colocalize with TRP73⁺ CR cells (yellow arrows). Scale bars = 25 µm. (*C*) Quantification of CR cell density, total EdU⁺ cell number, and EdU⁺ CR cell number along the hippocampal fissure. Each data point represents one animal, with 2–3 brain sections analyzed per animal and treated as nested observations. Statistical analysis was performed using nested *t*-tests.

**Figure S4**


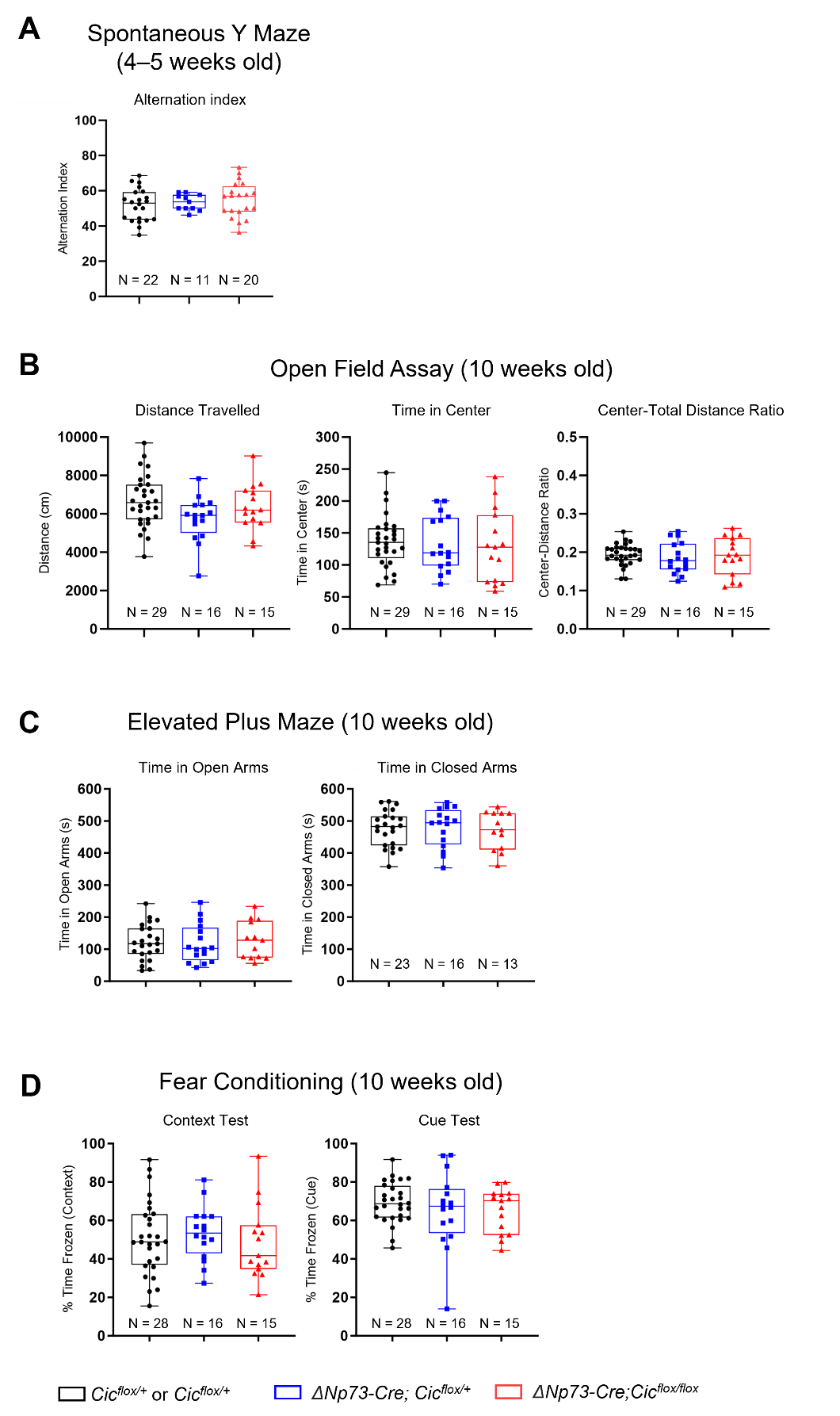


**Figure S4. Selective loss of CIC from Cajal-Retzius cells does not affect specific behaviors.** (*A*) 4- to 5-week-old *Cic^flox^*, *ΔNp73-Cre; Cic^flox/+^*; and *ΔNp73-Cre; Cic^flox/flox^* mice were subject to the spontaneous Y maze assay. 10-week or older *Cic^flox/+^*, *ΔNp73-Cre; Cic^flox/+^*; and *ΔNp73-Cre; Cic^flox/flox^* mice were subject to (*B*) the open field assay, (*C*) the elevated plus maze, and (*D*) the fear conditioning assay. Data are presented as box and whisker plots with all data points shown. Each data point represents a single animal. Statistical analysis was performed using a one-way ANOVA.

**Figure S5**


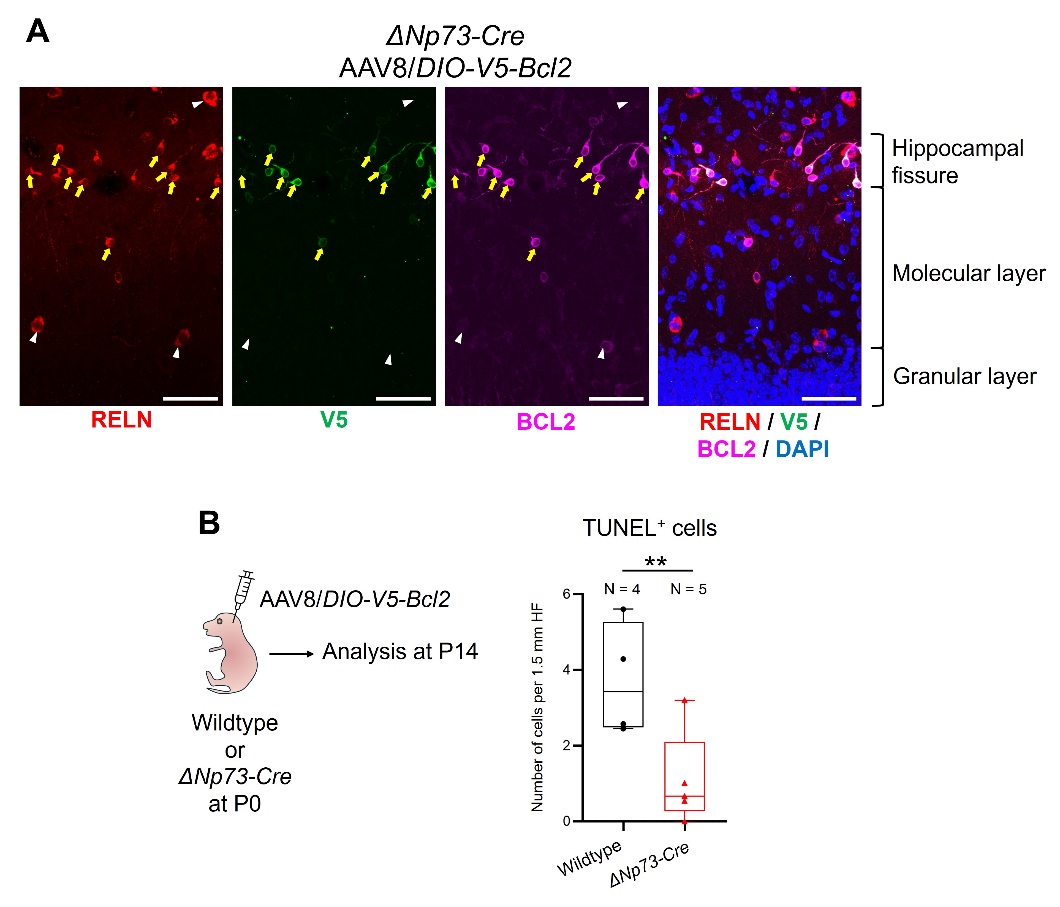


**Figure S5. BCL2 overexpression in CR cells reduces apoptotic cells.** (*A*) Representative images of CR cells along the hippocampal fissure from P20 *ΔNp73-Cre* mice injected with AAV8/*DIO-V5-Bcl2* at P0. Viral transduction efficiency and specificity is validated by colocalization of RELN, V5, and BCL2*.* Scale bars = 50 µm. (*B*) *Left*: Schematic of the experimental timeline for intracerebroventricular injection of wild-type and *ΔNp73-Cre* mice with AAV8/*DIO-V5-Bcl2*. *Right*: Quantification of TUNEL⁺ cells in the hippocampal fissure. Data are presented as box-and-whisker plots with all data points shown. Each data point represents one animal, with 4–5 brain sections analyzed per animal and treated as nested observations. Statistical analysis was performed using nested *t*-tests. **, *p* < 0.01.

**Figure S6**

**
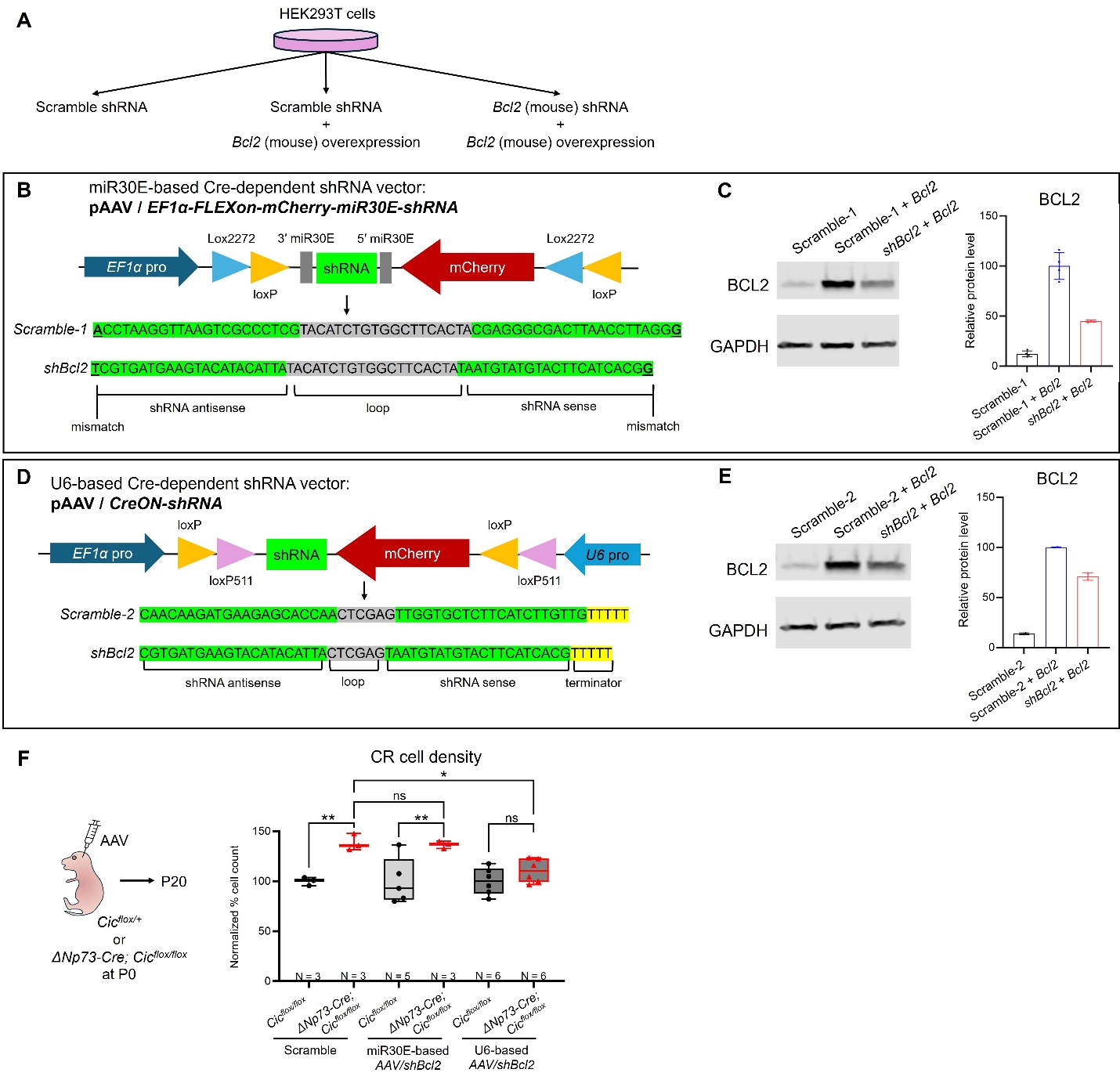
**

**Figure S6. Design and testing Cre-dependent *shBcl2*.** (*A*) Schematic of the *in vitro* assay: HEK293T cells were co-transfected with combinations of scramble shRNA, *shBcl2*, and *Bcl2* overexpression plasmids. (*B*) Diagram of the miR-30E-based Cre-dependent shRNA construct (pAAV/*EF1α-FLEXon-mCherry-miR30E-shRNA*). (*C*) Immunoblot analysis of BCL2 expression, with GAPDH as a loading control. Quantification is shown at right. Data are presented as scatter plots with mean ± SD; each data point represents one biological replicate. (*D*) Diagram of the U6-based Cre-dependent shRNA construct (pAAV/*CreON-shRNA*) and results of *in vitro* testing. Quantification is shown at right. Data are presented as scatter plots with mean ± SD; each data point represents one biological replicate. (*F*) *Left*: Overview of the *in vivo* experimental paradigm. *Right*: Quantification of normalized CR cell density along the hippocampal fissure in P20 mice. Data from knockout mice were normalized to littermate controls injected with the same virus to minimize variation between litters and viral preparations. Data are presented as box-and-whisker plots with all data points shown. Each data point represents one animal, with three brain sections analyzed per animal and treated as nested observations. Statistical analysis was performed using nested one-way ANOVA followed by Sidak’s *post hoc* test. *, *p* < 0.05; **, *p* < 0.01; ns, not significant.

**Figure S7**

**
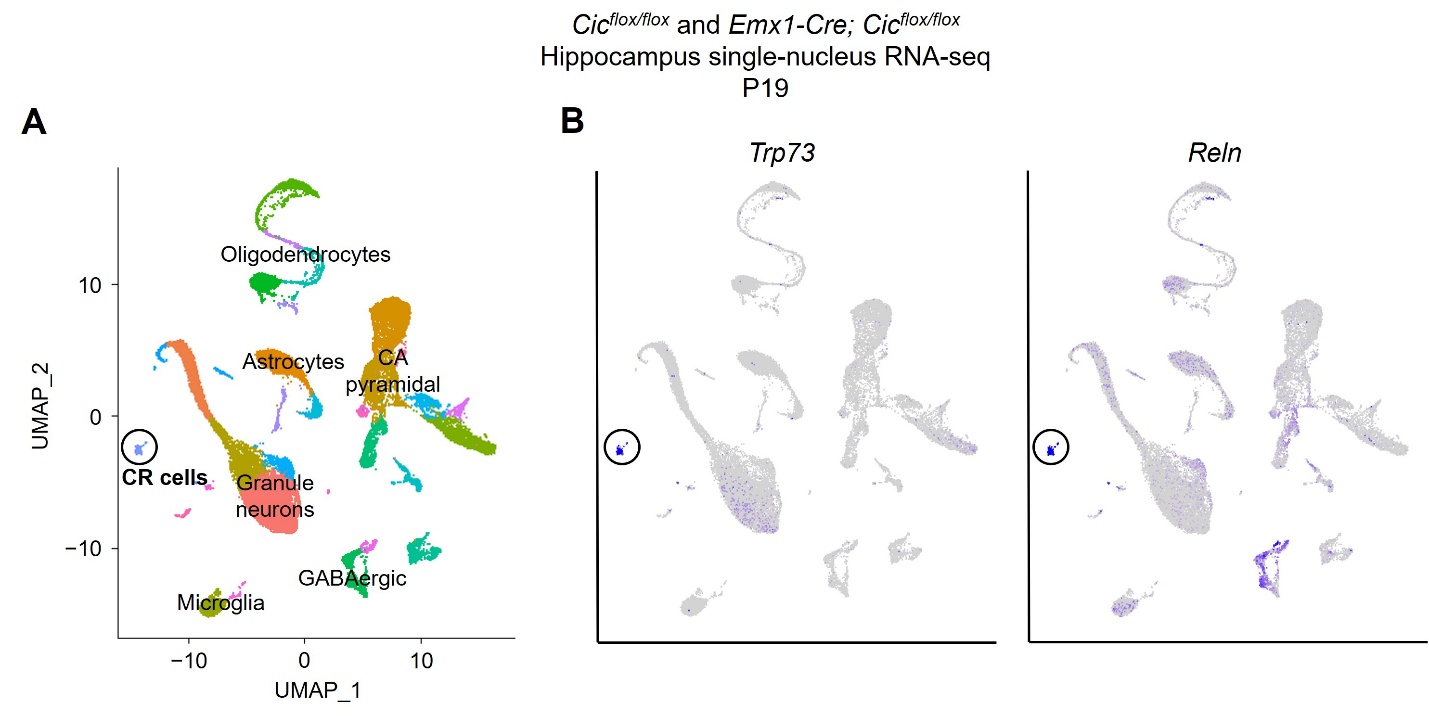
**

**Figure S7. Single-nucleus RNA sequencing of P19 control and *Emx1-Cre; Cic^flox/flox^* hippocampi.** (*A*) UMAP plot of hippocampal cell type clusters, with the Cajal-Retzius (CR) cell cluster circled. (*B*) UMAP feature plots showing *Trp73* and *Reln* expression marking the CR cell cluster.

**Figure S8**

**
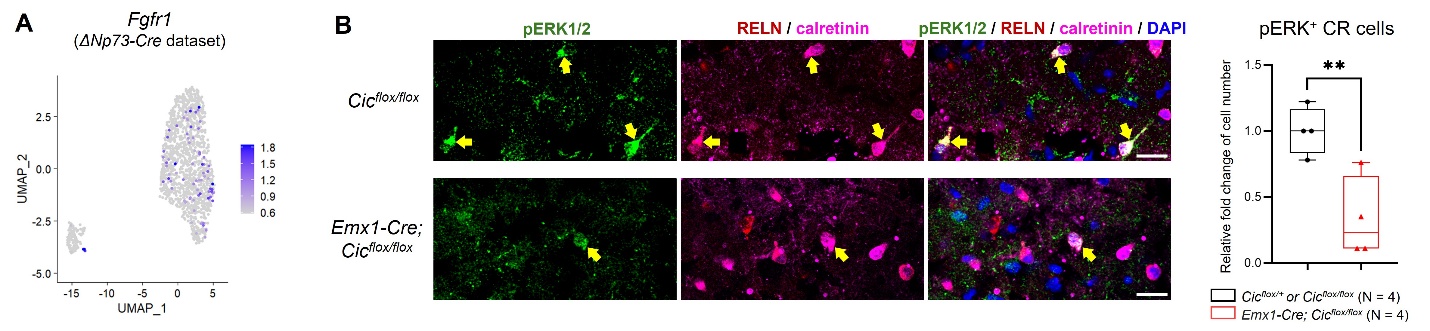
**

**Figure S8. Examination of FGF signaling components in *Cic* knockout mice.** (*A*) UMAP feature plot from the *ΔNp73-Cre; Cic^flox/flox^; tdT* scRNA-seq dataset showing *Fgfr1* expression in a subset of Cajal-Retzius (CR) cells. (*B*) Representative images of pERK1/2 immunoreactivity in CR cells along the hippocampal fissure of *Cic^flox/flox^* and *Emx1-Cre; Cic^flox/flox^* mice at P11. Yellow arrows indicate pERK1/2⁺ RELN⁺ calretinin⁺ CR cells. Scale bars = 20 µm. Quantification of the relative fold change in pERK⁺ CR cell number is shown at right. Data are presented as box-and-whisker plots with all data points shown. Each data point represents one animal. Statistical analysis was performed using Welch’s *t*-test. **, *p* < 0.05.
