# Supplementary figures and images for: Capicua regulates the survival of Cajal-Retzius cells in the postnatal hippocampus
Source: Cell Death Dis. 2025 Dec 22;16(1):898. doi: 10.1038/s41419-025-08206-7 (PMC12722426; doi:10.1038/s41419-025-08206-7)

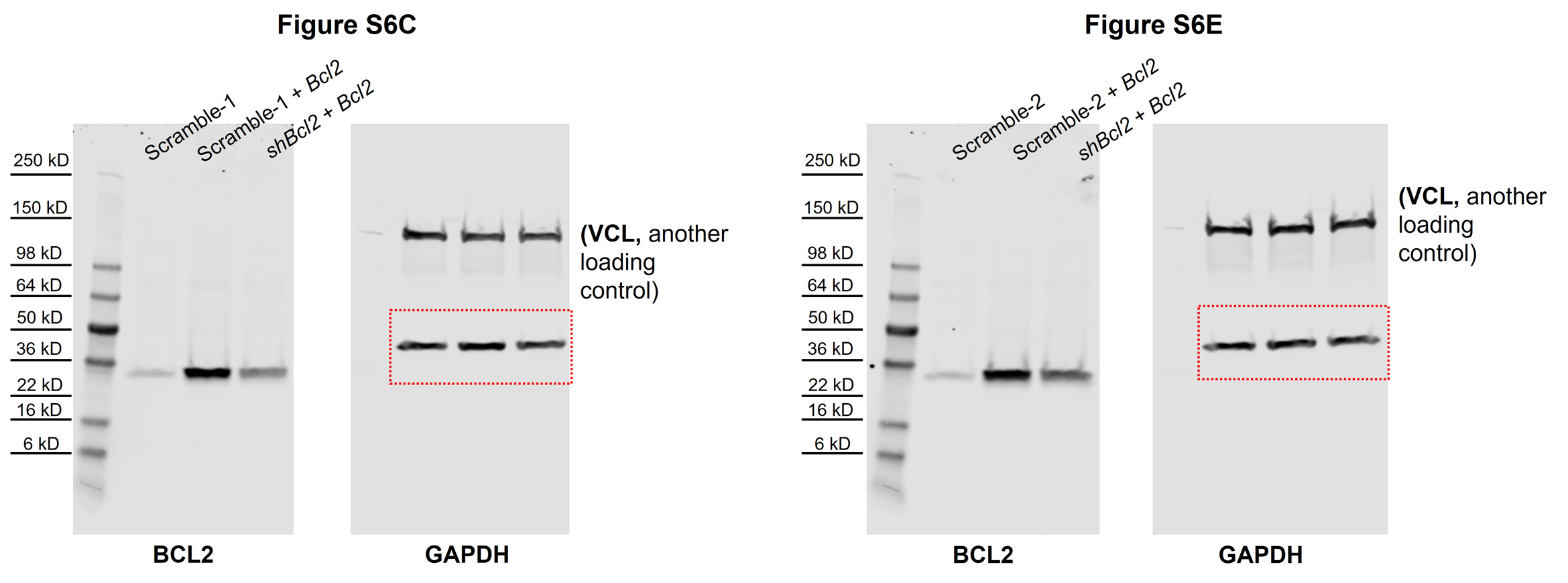

Supplement: Supplementary file 2 — Original western blots [file 41419_2025_8206_MOESM2_ESM.tif]
